# Capturing women’s bodily experiences: Conception and formative stages of the women’s somatic experience inventory

**DOI:** 10.1371/journal.pone.0353167

**Published:** 2026-07-13

**Authors:** Kelsey L. Piersol, Amanda P. Colangelo, Jennifer F. Buckman

**Affiliations:** Department of Kinesiology and Health, Rutgers University–New Brunswick, New Jersey, United States of America; New York University Abu Dhabi, UNITED ARAB EMIRATES

## Abstract

Assessment of women’s bodily experiences is used primarily to characterize negative psychological features of menstruation. However, women’s somatic states span a full spectrum of valence, emanate from multiple body systems, and affect physical and psychological well-being every day, throughout life and a more complete understanding of these states is important for the advancement of the science of women. This study presents the conception and formative stages of a comprehensive inventory about women’s somatic states, bodily experiences, and physical perceptions to be called the Women’s Somatic Experience Inventory. Three hundred biologically female university students (ages 18–25) rated the frequency and valence of diverse somatic experiences. An iterative approach was used: a systematic review compiled somatic items, variable clustering techniques reduced items, and confirmatory factor analysis and structural equation modeling guided factor refinement and proof-of-concept. Nine unpleasant latent factors (Kinesthetic Disconnect, Generalized Pain, Gastrointestinal Distress, Illness, Low Energy, Neurological Discomfort, Metabolic Stress, Heat & Urgency Response, and Sympathetic Activation) and four pleasant latent factors (Physical Alignment, Physical Intimacy, Activated & Attractive, and Parasympathetic Activation) were identified. Validated surveys of premenstrual symptoms and body awareness, use of hormonal contraception, and exercise and alcohol use behaviors were associated with pleasant and unpleasant factors, serving as initial proof-of-concept. These results support further refinement of the factors to be included in the inventory. With further testing and validation, the identified 73 somatic items and 13 factors could form the Women’s Somatic Experience Inventory, the first comprehensive inventory for understanding women’s somatic experiences, how these experiences change across female reproductive life stages, and how they interact with lifestyle behaviors.

## Introduction

Human behavior is influenced by a multitude of conscious and subconscious factors that reflect the processing of intero- and exteroceptive information. Over two decades ago, the somatic marker theory [1] posited that biologically-based body states are fundamental features of emotion (e.g., racing heart is associated with anxiety); that distinct profiles of somatic states become paired with events and experiences (e.g., intoxication mutes the racing heart); and that the valence of a somatic state directly affects decision-making (e.g., reducing feelings of anxiety by drinking). Recently, research into the role of interoception in behavioral choices has grown exponentially. This research creates a theoretical framework for understanding how body sensations and perceptions of body sensations impact psychological well-being and health behaviors [2,3].

The somatic marker hypothesis posits that a neural map of body state exists and that homeostatic deviations produce neural signatures that elicit corrective responses (i.e., allostatic processes) that promptly return the body to homeostasis [1]. Larger homeostatic deviations result in larger corrective responses and engage more neural resources to ‘mark’ a given body state with a consciously perceived ‘feeling.’ Stronger feelings require the individual to attend to that feeling and take behavioral action to correct it [4]. This ability to detect, perceive, and emotionally tag somatic deviations, also referred to as interoception [2,5], drives decision making and behavioral choices. However, to our knowledge, no comprehensive woman-specific measure currently exists that assesses the salience, valence, frequency, and dynamics of women’s somatic states, bodily experiences, and physical perceptions.

Within the field of women’s health, much of the existing research on somatic states and their measurement strategies focus on negative aspects, especially regarding symptoms of the menstrual cycle (see [6] for a review). Longitudinal studies of female-specific somatic experiences take a disorder-centered, symptom-reliant approach and focus heavily on the unpleasant changes that can occur during the perimenstrual period [7]. This method can be useful when examining relations between menstrual symptoms and behaviors of clinical populations, such as those with premenstrual dysphoric disorder (PMDD) [8–10], but overlooks the daily bodily experiences of all women, especially experiences that positively impact well-being and behavior [11–14].

Although inventories of women-specific somatic experiences are lacking, the Body Awareness Questionnaire (BAQ) [15] was designed to assess physical experiences that align with subjectively-experienced somatic states. The BAQ measures sensitivity to normal, non-emotive body processes such as sensitivity to body rhythms and cycles, detection of abnormal functioning, and anticipation of bodily reactions. Higher BAQ scores reflect more awareness of bodily processes and comfort with normal bodily functioning, indicating greater interoceptive sensibility. It captures the salience and valence of bodily experiences by framing physiological states in relation to expectations from those states (e.g., “I can tell when I go to bed how well I will sleep that night.”). Individuals with poorer metabolic health report lower body awareness, whereas individuals with higher physical activity levels report higher body awareness [16–18]. The BAQ is not women-specific but has been used, with mixed results, in studies seeking to characterize differential experiences of menstruation [19–22], pregnancy [23,24], and menopause [25,26]. The present study seeks to develop a comparable scale that emphasizes states, experiences, and perceptions unique to women.

Among existing women-specific surveys, the primary focus is on fluctuation of ovarian hormones across the menstrual cycle and their ability to exert widespread and negative alterations in physiological states that manifest as potentially pathological changes in physical and emotional states [27,28]. The Premenstrual Symptoms Screening Tool (PSST) [29] is a two-part clinical tool that screens for premenstrual symptom disorders, such as Premenstrual Syndrome (PMS) and PMDD. Although intended as a clinical screening tool, the first 14 items of the PSST assess symptom severity across a series of mood, behavioral, and physical domains, with higher scores reflecting more severe premenstrual symptoms. The PSST groups physical symptoms into a single item, limiting the precision by which somatic sensations can be assessed. Thus, these items provide insight into a limited subset of female-specific somatic experiences and perceptions, namely those related to the menstrual cycle, that can be built upon to develop a more holistic women’s inventory [30–32].

This study sought to identify a comprehensive list of women-specific subjective physical experiences that span valences (unpleasant to pleasant), body systems (e.g., neural, musculoskeletal), and domains of state, experience, and perception. We then used an iterative process, driven by conceptual goals and quantitative clustering approaches, to refine the list of items from which factors were identified. Finally, as proof-of-concept, we tested the relationship of identified latent factors to two existing validated measures of body awareness and premenstrual symptoms and to lifestyle behaviors, including alcohol use, exercise behavior, and hormonal contraceptive use. We *a priori* hypothesized four distinct factors that captured subjective experiences across a full spectrum of valences: physical movement and proprioception (kinesthetic), metabolic state (energetic), body organs and senses (visceral), and general health (wellness). Following factor identification, we hypothesized that pleasant sensations would be endorsed more frequently than unpleasant sensations, and that unpleasant factors would be positively associated with premenstrual symptoms and negatively associated with body awareness; the opposite relationships were hypothesized for pleasant factors. Factor relationships with alcohol use, exercise, and hormonal contraceptive use were exploratory but generally posited that unhealthy behaviors (i.e., drinking and sedentary behaviors) would be positively associated with unpleasant factors and healthy behaviors would be positively associated with pleasant factors. Emphasis of this methodological study is on inventory refinement; further validation with different samples will be essential before use in research and applied settings.

## Methods

### Participants

Three hundred ten participants were recruited from Rutgers University via email listservs, social media platforms, flyers, and word-of-mouth. Eligibility was determined based on a brief online survey accessed using a link or QR code. Participants were deemed eligible if they were female with a regular menstrual cycle between 25–35 days, enrolled as a full-time university student, 18–25 years old, had no history of psychosis, were not pregnant or breastfeeding, and had no current physical conditions that required daily medication (except for hormonal contraception (HC)). Rutgers University’s Institutional Review Board approved this study for the protection of human subjects involved in research. Sample characteristics are shown in Table 1.

**Table 1:** Sample characteristics.

	*n* = 300
**Age** (yrs)	20.1 ± 1.3
**Race**	
Asian	40.0 (120)
Black	6.3 (19)
White	41.0 (123)
>1 race or Other	12.7 (28)
**Ethnicity**	
Non-Hispanic	86.3 (259)
Hispanic	13.7 (41)
**Hormonal Contraceptive Users**	39.0 (117)
**Premenstrual symptoms**	20.3 ± 9.4
**Body awareness**	73.3 ± 17.8
**Drinking recency**	
Never	20.8 (62)
Not within past 4wks	17.8 (53)
Within past 4wks	61.4 (183)
**Within past 4wks drinkers**	
Drink days	3.9 ± 3.0
# drinks	13.9 ± 13.0
Max drinks per episode	4.8 ± 2.9
**Exercise Participation**	
Cardio/Aerobic	84.0 (252)
Strength/Resistance	37.0 (111)
**Within Exercisers**	
Frequency (days per wk):	
Cardio/Aerobic	3.8 ± 1.7
Strength/Resistance	3.1 ± 1.4
Duration (min per session):	
Cardio/Aerobic	39.6 ± 14.9
Strength/Resistance	46.7 ± 14.8
Exposure (min per wk):	
Cardio/Aerobic	162.0 ± 107.4
Strength/Resistance	157.6 ± 99

*Note:* Mean ± standard deviation; % (*n*).

All participants provided digital informed consent prior to completing the screener and survey. Eligible participants were invited via an individualized emailed link to a Qualtrics survey. Email reminders were sent to prompt initiation and completion of the survey. Ten participants were excluded from analysis for either finishing <25% of the survey or for inputting the same answer for >75% of survey items; thus, the final sample was 300.

### Survey measures and design

The current survey included (a) demographics, health, and behavior surveys to assess participant characteristics; (b) supplemental validated surveys to illustrate factor qualities and ground findings in extant literature, and (c) a list of “somatic items”, which were words or brief phrases that depicted bodily states, physical perceptions, and sensory experiences, to assess the frequency and valence of somatic experiences. The identification, selection, and refinement of these items is presented in Results.

The survey used for the present analyses consisted of 4 blocks. Block 1 was first for all participants and included demographics, health history, reproductive health, exercise, and alcohol use questions. Basic demographics collected included age, year of university study, and race and ethnicity. A modified Reproductive Status Questionnaire [7] was used to categorize women who used HC and those who were naturally cycling. Exercise was assessed as frequency (typical days per week) and duration (minutes per day, in intervals: < 10 minutes, 10–20 minutes, 21–30 minutes, 31–40 minutes, 41–50 minutes, 51–60 minutes, and >60 minutes) of aerobic and resistance exercise, with an option to indicate “No exercise”. For the analyses, duration intervals were converted to numbers at the midpoint of each interval (<10 min = 5 min, 10–20 min = 15 min, 20–30 min = 25 min… > 60 min = 65 min). Final exercise variables included exercise status (i.e., exerciser or non-exerciser); aerobic exercise status (yes or no) and weekly exposure (minutes per week); and resistance exercise status (yes or no) and weekly exposure (minutes per week). Alcohol use was assessed as the last time they consumed more than a sip of alcohol (within the last week, 1–2 weeks ago, 3–4 weeks ago, more than a month but less than 3 months ago, more than 3 months ago, and never) to categorize “non-drinkers” (did not consume alcohol in the past 4 weeks) and drinkers (did consume alcohol in the past 4 weeks). Drinkers reported the number of standard drinks consumed and the number of hours drinking every day for the past 30 days using an online version of the Timeline FollowBack (TLFB; SMASH Labs, Providence, RI) [33]. Alcohol variables included past 30-day total frequency and quantity and the maximum number of drinks consumed in a single drinking day.

Blocks 2–4 each contained supplemental items from validated surveys and 50–70 newly identified somatic items (described below). Fourteen supplemental survey items were from the first section of the Premenstrual Symptoms Screening Tool (PSST) [29]. A sum score of all 14 items (“Not at all” (1), “Mild” (2), “Moderate (3), and “Severe” (4)) was created as a proxy measure of premenstrual symptom severity. The items showed excellent internal consistencies (α = .91). Higher PSST scores reflect more severe premenstrual symptoms. Eighteen supplemental survey items were from the Body Awareness Questionnaire (BAQ) [15]. Items were scored on a 7-point scale, from 1, “Not at all true of me” to 7, “Very true of me” and summed to create a total score of body awareness. The items showed strong internal consistencies (α = .87). Higher BAQ scores reflect more awareness of bodily processes and comfort with normal bodily functioning. In addition, participants were presented with a total of 204 somatic items distributed across Blocks 2–4. They rated the frequency that they experienced each somatic item in the past 30 days on a 5-point Likert scale: “Never” (0), “Rarely” (1), “Sometimes” (2), “Most of the time” (3), or “Always” (4). On the next screen, participants were asked to provide valence ratings on a 7-point Likert scale, from “Very unpleasant” (−3) to “Very pleasant” (+3), with 0 being “Neither unpleasant nor pleasant”. Participants also had the option to rate the item as “Unclear” (not scored) if they could not imagine what an item would feel like or if the meaning of the item was unclear; these items were not rated for valence. Items endorsed as “Never” (in past 30 days) were included in valence ratings as they might have been experienced outside of the past month (e.g., rare or acute events, such as being sick).

Blocks 2–4 were counterbalanced across participants, and somatic items within blocks were randomized. Prior to each block, a brief introduction was provided that reminded participants that the somatic items were describing physical sensations, that they should answer based on their own personal physical experiences of their body, and that there were no right or wrong answers. In each block, subjective experience of each somatic item was assessed across two domains. All participants rated 1) frequency and 2) valence of each somatic item. Participants were compensated with an Amazon gift card worth $5 for each block of the survey they completed, up to $20.

### Data analysis

#### Item refinement and clustering with PROC VARCLUS.

We used PROC VARCLUS in SAS 9.4 (SAS Institute, Cary, NC) based on data structure and goal similarities with the Multidimensional Assessment of Interoceptive Awareness (MAIA) [34] and MAIA-II [35]. This methodology is preferrable over other exploratory data reduction tools (e.g., exploratory factor analysis) during survey development stages as it superior when there are a large number of items and discrete, iterative pruning steps are needed to characterize theoretically-sound clusters. Because PROC VARCLUS cannot accommodate missing data, the data first underwent multiple imputation of maximum likelihood estimates using the EM algorithm within SAS PROC MI. Imputation was performed after initial conceptual item reduction steps were employed.

PROC VARLCUS is a multi-step clustering method that utilizes oblique principal components analyses (PCA) to group items into clusters based on their correlation matrices. All items begin in a single cluster and may be split into two clusters if the Eigenvalue is the highest second Eigenvalue and greater than a set cutoff (the default is 1). Items in the target cluster undergo PCA with a QUARTIMAX rotation to create two principal components that maximize intra-cluster correlations and minimize inter-cluster correlations. Items within the original cluster are then assigned to the component with which it is most highly correlated. Every step attempts to maximize the amount of variance explained; each item is tested to see if reassignment to a different cluster increases the variance explained. Reassignment can occur at any step. If an item is reassigned, the components are recomputed prior to testing the next item. The process repeats until the stopping rule is met (i.e., Eigenvalue is ≤ 1) or the specified number of clusters is created (by using the “MAXCLUSTERS=” option).

The frequency of each item within the unpleasant and pleasant datasets were input (separately) into PROC VARCLUS analyses to extract first-order clusters using the MAXCLUSTERS option, with the number of clusters based on a balance of quantitative item fit and theoretical interpretation. The number of clusters was increased until most items within a cluster had an R^2^ of ~0.5 and appeared to have a conceptually unifying theme. Second-order clusters were then created using PROC VARCLUS on first-order clusters to enhance variable reduction and factor identification. The correlation matrix of first-order cluster models was input to PROC VARCLUS, with a goal of R^2^ > 0.5 for each first-order cluster within a second-order cluster.

For follow-up analyses of second-order cluster correlations between and within valence, the original, non-imputed frequency data were used. For each second-order cluster, a sum score was computed for each participant (13 total scores per participant; 9 unpleasant and 4 pleasant). Scores were created by summing the coded frequency ratings of each item within each second-order cluster (Never = 0; Rarely = 1; Sometimes = 2; Most of the time = 3; Always = 4). Higher sum scores indicated they reported experiencing items more frequently than lower scores; this could mean higher frequency ratings on a few items within the cluster, mid-level ratings across all items, or higher frequency ratings across all items. Only complete cases were used within each second-order cluster sum score. Pearson correlational analyses were used to examine relationships among second-order clusters, within and between valences, and guide decisions for Confirmatory Factor Analysis. Alpha was set at 0.05.

#### Validation with confirmatory factor analysis.

Second-order clusters were confirmed and refined with Confirmatory Factor Analysis performed using MPlus version 8.11 [36]. Separate CFAs were performed for unpleasant and pleasant latent factors using the nine and four second-order cluster solutions from PROC VARCLUS, respectively. Significant correlations among second-order clusters within valence guided the decision to correlate all latent factors in each of the two CFAs. Maximum likelihood estimation was used to handle missing data. Item loadings from STDYX were examined. Modification indices with values >15 guided the stepwise removal of four unpleasant items and four pleasant items; model fit was noted at each step by examining the root mean square error of approximation (RMSEA), comparative fit index (CFI), Tucker-Lewis Index (TLI), and standard root mean square residual (SRMR). Only modifications that improved model fit were retained. As in the MAIA scale development, we sought a final model with “good” fit in at least two of the following: RMSEA (≤.06), CFI and TLI (≥.95), and SRMR (≤.08) [37–39]. Qualities of the latent factors from the final models of unpleasant and pleasant items were examined and described using the raw frequency ratings (e.g., frequency means by factor, range of frequency ratings). Global unpleasant and pleasant scores were created by taking the average of all unpleasant and pleasant factor frequency ratings, with higher scores implying more frequent experiences. An exploratory *t*-test examined differences between global scores.

#### Proof of concept with structural equation modeling.

The relationships of the latent factors from the CFA to psychometrically validated surveys of premenstrual symptoms and body awareness were assessed with structural equation modeling (SEM). Similarly, SEM was used to examine the relationships of the latent factors with HC status and behavioral measures of exercise and alcohol use. Separate SEMs were performed for each variable. The models assessing weekly exposures to aerobic (*n* = 252) and resistance (*n* = 111) exercise were performed only within participants who performed that type of exercise. Similarly, alcohol frequency, quantity, and maximum drinks per episode were assessed using only past 30-day drinkers (*n* = 183). SEM analyses were not corrected for multiple testing due to the exploratory nature of this study.

## Results

### Somatic item identification and refinement

An extensive literature review was completed to identify somatic states related to general physical health as well as those related to menstrual health and substance use. The review leveraged existing survey banks (e.g., NIH Toolbox, HealthMeasures, PROMIS), PubMed and GoogleScholar searches, and general internet searches of somatic descriptors (e.g., words or phrases used in somatic therapy or Eastern medicinal techniques). Search terms included words and phrases, such as “somatic”, “physical sensations”, “bodily experiences”, “intoxication”, “menstrual symptoms”, “hangover”, “interoception”, “proprioception”, and “body awareness”. Somatic sensations across a wide number of body systems and physiological experiences that would, theoretically, rate along a full valence spectrum from very unpleasant to very pleasant, were identified. An initial list of 300 items was compiled (see S1 Appendix for full list).

Variable reduction and selection were iterative using both data- and theory-driven processes. First, a pre-data conceptual item reduction step culled the original 300 items to 204 by removing synonymous terms and items with multiple/unclear interpretations. This step was supported by 5 female undergraduate research assistants in the laboratory who were not involved in the item selection for or the development of the survey. These individuals were presented with a list of items and asked to identify, from their perspective, highly synonymous terms, confusing terminology (i.e., words they were unfamiliar with or words that had multiple/unclear interpretations), and terms they considered scientific or medical jargon.

A post-data conceptual item reduction step was undertaken to align items with the initial goals of the project: to create a comprehensive measure that covered multiple body systems and somatic experiences of young women, with emphasis on the ability to measure changes associated with the menstrual cycle and/or behaviors, such as alcohol use or exercise. Low endorsement items and unclear items were removed using frequency ratings from the total sample, and from the non-drinker and drinker subsamples (because alcohol use can create specific somatic experiences (e.g., spinning, high, lips numb, body rush)). Twenty items were trimmed based on ≥20% of both subsamples rating the item as “Unclear”. Four words were trimmed based on being reported as “Never” by 61% of the full sample. Five duplicates were removed. This initial variable reduction concluded with 175 somatic items for imputation and further analyses.

Mean valence was then computed for each item using data from the total sample. Items with an average valence <0 (*n* = 120) were included in the “unpleasant” dataset; items with an average valence >0 (*n* = 55) were included in the “pleasant” dataset. This parallels the Positive and Negative Affect Schedule [40] and ensured that both unpleasant and pleasant items were maintained.

### Item clustering and validation

#### Unpleasant items.

A data-driven item reduction step was completed via several iterations of PROC VARCLUS on the 120 unpleasant items, which allowed observation of clustering patterns and identification of items that consistently fit poorly within a cluster. Cluster number was maximized at a 53-cluster solution. The goal was to allow the procedure to quantitatively group items that could be considered synonymous into their own clusters to eliminate items that were very close in meaning and interpretation. Each cluster consisted of 1–4 items and were examined for synonyms and themes. When a synonym was identified (e.g., nauseous, nauseated), the synonymous item that had the lower percent of “Unclear” ratings or greater percentage of endorsement above “Never” was maintained. Thirty items were dropped during this stage. Additional iterations of PROC VARCLUS were performed to identify items that did not load well onto clusters or were frequently in their own cluster; items were removed (n = 10) unless they were considered as critical to the survey’s purpose (e.g., tender breasts). Iterations continued until quantitative fit (R^2^ > .5) and theoretical interpretation were maximized. The final first-order model of unpleasant items consisted of 30 first-order clusters of 80 items.

Second-order clustering of unpleasant items was then performed to tap into dimensional grouping of first-order clusters to be considered as latent factors. The selected model identified 14 second-order clusters. Five second-order clusters contained only one first-order cluster and were not considered in the factors, which eliminated 8 items; the single cluster containing “tender breasts” was maintained. A post hoc conceptual refinement step eliminated 19 additional items for the following reasons: redundancy, having emotional or cognitive interpretations, or misalignment with conceptual factor themes. For redundant items, the original frequency data were referenced and the items with the least percentage of unclear ratings and greatest endorsements above “never” were kept; 5 redundant items were removed. Items determined to have substantive emotional or cognitive interpretations (n = 6) were culled. Finally, after identification of the unifying theme and naming of each second-order cluster as a factor, items that did not seem to align with the conceptual theme of its second-order cluster were also removed (n = 8).

The best fitting second-order model for unpleasant items included 9 second-order clusters that included 53 items (Table 2). The clusters were examined for overarching themes and named based on the unifying theme across items: Sympathetic Activation, Generalized Pain, Gastrointestinal (GI) Distress, Illness, Kinesthetic Disconnect, Low Energy, Neurological Discomfort, Metabolic Stress, and Heat & Urgency Response. Sum scores from unpleasant clusters were all significantly correlated with one another, with correlation coefficients ranging from 0.25–0.68 (S1 Appendix, Table 1). Illness had the weakest correlations with other unpleasant clusters (*r* = 0.25–0.41).

**Table 2:** Unpleasant item cluster identification and factor validation.

		PROC VARCLUS			FACTOR ANALYSIS		
First-Order Cluster #		R^2^ - 1^st^ Order Cluster	R^2^ - 2^nd^ Order Cluster	Mean Valence Ratings	Estimate	Standard Error	Residual variance
**Sympathetic Activation**							
Cluster 3	Rapid breath	0.58	0.88	−1.2 ± 0.8	0.69	0.04	0.53
	Faint	0.6		−1.5 ± 0.9	0.69	0.04	0.52
	Breathless	0.59		−1.4 ± 0.9	0.7	0.04	0.51
Cluster 24	Racing heart	0.68	0.71	−1.4 ± 1.0	0.57	0.04	0.68
	Heart palpitations	0.68		−1.6 ± 1.1	–	–	–
Cluster 13	Sudden thirst	0.61	0.47	−0.9 ± 0.8	0.56	0.05	0.69
	Dry mouth	0.64		−1.3 ± 0.8	0.5	0.05	0.75
Cluster 30	Tunnel vision	0.7	0.69	−0.9 ± 1.1	0.48	0.05	0.77
**Generalized Pain**							
Cluster 4	Muscle pain	0.63	0.72	−1.3 ± 0.9	0.48	0.05	0.77
	Joint pain	0.65		−1.6 ± 0.9	–	–	–
	Achy	0.64		−1.4 ± 0.9	0.67	0.04	0.55
Cluster 11	Headache	0.73	0.76	−1.9 ± 0.9	0.65	0.04	0.57
	Nauseous	0.73		−2.0 ± 1.0	0.68	0.04	0.54
Cluster 12	Abdominal cramp	0.58	0.52	−2.0 ± 1.0	–	–	–
	Lower abdominal pain	0.72		−2.0 ± 1.0	0.49	0.05	0.76
Cluster 20	Tender breasts^a^	1	—	−1.0 ± 0.9	0.46	0.05	0.79
**Gastrointestinal Distress**							
Cluster 25	Constipated	0.69	0.76	−1.9 ± 0.9	0.54	0.05	0.71
	Diarrhea	0.69		−2.1 ± 1.0	0.57	0.05	0.68
Cluster 26	Upset Stomach	0.7	0.76	−1.9 ± 0.9	0.63	0.05	0.61
	Gassy	0.7		−1.4 ± 1.0	0.62	0.05	0.62
**Illness**							
Cluster 2	Cough	0.58	0.8	−1.4 ± 0.8	0.67	0.04	0.56
	Sick	0.75		−2.2 ± 0.9	0.72	0.04	0.48
	Sore throat	0.52		−1.6 ± 1.0	0.66	0.04	0.57
Cluster 15	Runny nose	0.76	0.8	−1.4 ± 0.8	0.62	0.05	0.62
	Stuffy nose	0.76		−1.5 ± 0.9	0.59	0.05	0.65
**Kinesthetic Disconnect**							
Cluster 1	Restless	0.55	0.82	−1.5 ± 1.0	–	–	–
	Unstable	0.64		−1.7 ± 1.0	0.66	0.04	0.56
Cluster 16	Decreased sexual enjoyment	0.66	0.45	−1.3 ± 1.0	0.38	0.06	0.85
	Numb	0.66		−1.2 ± 1.0	0.55	0.05	0.7
Cluster 17	Fidgety	0.62	0.65	−1.0 ± 0.8	0.62	0.04	0.61
Cluster 18	Tense	0.57	0.71	−1.6 ± 0.9	0.58	0.04	0.67
Cluster 21	Weak	0.56	0.85	−1.5 ± 1.0	0.66	0.09	0.57
	Uncomfortable	0.62		−1.8 ± 1.0	0.71	0.03	0.5
**Low Energy**							
Cluster 7	Bloated	0.59	0.62	−1.8 ± 0.9	0.56	0.05	0.68
	Heavy	0.59		−1.5 ± 1.0	0.62	0.04	0.62
Cluster 10	Fatigued	0.67	0.66	−1.8 ± 0.9	0.72	0.03	0.48
	Sleepy	0.65		−1.1 ± 1.1	0.53	0.05	0.72
Cluster 28	Slouchy	0.73	0.81	−1.1 ± 0.7	0.59	0.04	0.66
	Sluggish	0.73		−1.4 ± 0.8	0.7	0.04	0.51
**Neurological Discomfort**							
Cluster 6	Head spinning	0.72	0.82	−1.6 ± 1.0	0.76	0.03	0.42
	Dizzy	0.72		−1.5 ± 1.0	0.77	0.03	0.41
Cluster 22	Pins and needles	0.62	0.82	−1.1 ± 1.0	0.55	0.05	0.7
	Tingly	0.59		−0.5 ± 1.1	0.58	0.04	0.66
**Metabolic Stress**							
Cluster 27	Spinning	0.71	0.82	−1.4 ± 1.0	0.66	0.04	0.56
	Feverish	0.71		−1.7 ± 1.0	0.57	0.05	0.67
Cluster 29	Shaky	0.65	0.82	−1.2 ± 0.9	0.69	0.04	0.52
	Hunger pang	0.59		−1.1 ± 0.9	0.58	0.05	0.66
	Burning in stomach	0.56		−1.7 ± 0.9	0.55	0.05	0.7
**Heat & Urgency Response**							
Cluster 8	Overheated	0.43	0.68	−1.6 ± 1.0	0.62	0.05	0.62
	Sudden urge to defecate	0.58		−1.3 ± 1.1	0.55	0.06	0.7
	Sudden urge to urinate	0.57		−1.3 ± 1.0	0.58	0.05	0.66
Cluster 19	Warm limbs	0.67	0.68	−0.2 ± 1.1	0.46	0.06	0.79
	Cheeks warm	0.67		−0.2 ± 1.0	0.43	0.06	0.81

*Note:* Clusters were identified using PROC VARCLUS and verified as latent factors with Confirmatory Factor Analysis (CFA). Dashed lines denote items removed from CFA due to high modification indices. ^a^Tender breasts was directly relevant to study purpose and manually selected for inclusion in Generalized Pain due to fitting in several prior models of the second-order pain cluster.

**Fig 1:**
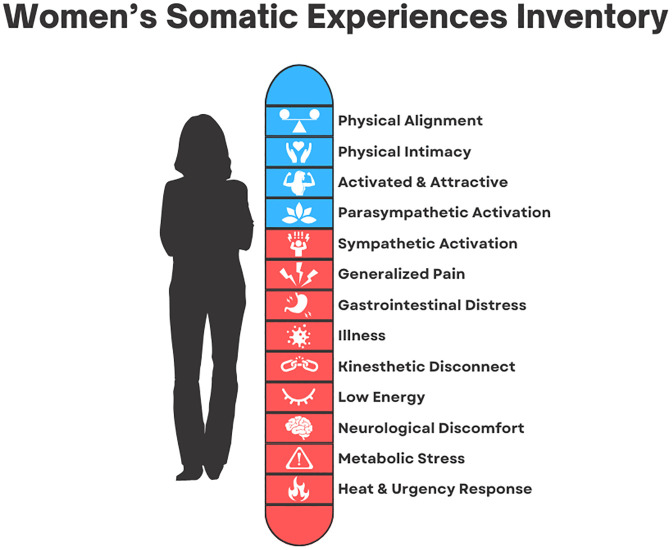
Graphic of the identified pleasant (in blue; Physical Alignment, Physical Intimacy, Activated & Attractive, and Parasympathetic Activation) and unpleasant (in red; Sympathetic Activation, Generalized Pain, Gastrointestinal Distress, Illness, Kinesthetic Disconnect, Low Energy, Neurological Discomfort, Metabolic Stress, and Heat & Urgency Response) latent factors.

A CFA was then performed and unpleasant items with modification indices > 15 were removed. These included “heart palpitations”, “joint pain”, “abdominal cramping”, and “restless”. The final 9-latent factor model for the 49 unpleasant items (S2 Appendix) showed good model fit based on RMSEA and SRMR but CFI and TLI were below the threshold for a “good” fit (S1 Appendix, Table 2); all item loadings were significant (Table 2), and all latent factors were intercorrelated. The latent factors are illustrated in Fig 1.

#### Pleasant items.

Fewer positively-valenced items were identified from the literature and included in the survey. Thus, item reduction of the 55 pleasant items was more conservative than with unpleasant items to retain as many items as possible. Criteria for removal during the data-driven item reduction steps were similar to unpleasant items regarding synonyms and loadings; 17 items were removed during this stage.

The first-order cluster solution contained 18 clusters of 39 items. The correlation matrix from these 18 clusters were input into PROC VARCLUS to obtain a final model of 5 second- order clusters (Table 3); one single-item first-order cluster (smooth skin) was excluded. Like the final unpleasant clusters, the final 4 pleasant clusters were subjected to a post hoc conceptual refinement step to address item redundancy, non-physical interpretation, and conceptual fit. For redundant items, the item with the lowest “unclear” rating or the highest non-never frequency rating was maintained; 7 were removed. Finally, after identifying the unifying theme and naming each second-order cluster as a latent factor, items that did not seem to align with the theme of its second-order cluster were also removed (n = 3).

**Table 3:** Pleasant item cluster identification and factor validation.

		PROC VARCLUS				FACTOR ANALYSIS		
First-Order Cluster #		R^2^ - 1^st^ Order Cluster	R^2^ - 2^nd^ Order Cluster	Mean Valence Ratings		Estimate	Standard Error	Residual variance
**Physical Alignment**								
Cluster 1	Grounded	0.74	0.62	1.4 ± 1.3		0.55	0.05	0.7
Cluster 10	Coordinated	0.69	0.58	1.6 ± 1.1		0.55	0.05	0.7
Cluster 17	Stable	0.73	0.8	1.7 ± 1.2		0.72	0.04	0.48
Cluster 12	Agile	0.66	0.28	1.1 ± 1.2		0.4	0.06	0.84
	Flexible	0.66		1.4 ± 1.1		0.48	0.05	0.77
**Physical Intimacy**								
Cluster 2	Heightened sexual interest	0.79	0.77	1.3 ± 1.2		0.72	0.04	0.48
	Increased sexual enjoyment	0.79		1.7 ± 1.2		0.78	0.04	0.39
Cluster 6	Not bloated	0.66	0.67	1.8 ± 1.3		–	–	–
	Smooth digestion	0.66		1.6 ± 1.2		–	–	–
Cluster 18	Wanting physical contact	0.67	0.76	0.3 ± 1.5		0.52	0.05	0.73
	Full breasts	0.67		0.3 ± 1.4		0.51	0.06	0.74
**Activated & Attractive**								
Cluster 3	Good hair	0.5	0.69	2.1 ± 0.9		0.45	0.05	0.8
	Powerful	0.6		1.9 ± 1.1		0.59	0.05	0.65
	Strong	0.71		1.9 ± 1.0		–	–	–
Cluster 9	Centered	0.72	0.43	1.3 ± 1.1		0.5	0.05	0.75
Cluster 14	Energetic	0.58	0.79	1.9 ± 0.9		0.55	0.05	0.69
	Radiant	0.62		1.8 ± 1.1		0.7	0.04	0.52
	Resilient	0.6		1.7 ± 1.1		0.59	0.05	0.66
Cluster 8	Physically attractive	0.79	0.33	2.1 ± 1.2		0.46	0.05	0.79
**Parasympathetic Activation**								
Cluster 5	Hydrated	0.54	0.66	2.0 ± 1.1		0.6	0.04	0.64
	Calm breath	0.66		1.5 ± 1.1		0.6	0.04	0.64
Cluster 7	Clean	0.74	0.6	2.3 ± 1.0		0.5	0.05	0.75
	Well groomed	0.74		2.1 ± 1.0		–	–	–
Cluster 11	Balanced	0.56	0.83	1.6 ± 1.1		0.69	0.04	0.53
	Comfortable	0.65		2.1 ± 1.0		0.59	0.04	0.65
	Healthy	0.52		2.4 ± 1.0		0.65	0.04	0.57
Cluster 16	Rested	0.61	0.61	2.2 ± 1.1		0.62	0.04	0.61
	Relaxed	0.67		2.2 ± 1.0		0.61	0.04	0.63

*Note:* Clusters were identified using PROC VARCLUS and verified as latent factors with Confirmatory Factor Analysis (CFA). Dashed lines denote items removed from CFA due to high modification indices.

The best-fitting model for the pleasant items identified 4 second-order clusters of 28 items named based on conceptual similarities: Physical Alignment, Physical Intimacy, Activated & Attractive, and Parasympathetic Activation. Sum scores of pleasant clusters were all significantly correlated with one another (*r* = 0.40–0.64) (S1 Appendix, Table 3). Most of the correlations were moderate to strong.

A CFA was then performed and pleasant items with modification indices > 15 were removed. This included 4 items: “not bloated”, “smooth digestion”, “strong”, and “well groomed”. The final four-latent factor model for 24 pleasant items (S2 Appendix) demonstrated acceptable model fit based on RMSEA, SRMR, and CFI; TLI was 0.89 (S1 Appendix, Table 2). All item loadings were significant, and latent factors were intercorrelated (Table 3).

### Cluster correlations between valences

Correlational analyses were performed on the frequency sum scores of second-order clusters from PROC VARCLUS to examine relationships between unpleasant and pleasant clusters. Most correlations between unpleasant and pleasant clusters were not significant, and weak or close to 0 (Table 4). However, Parasympathetic Activation was significantly negatively correlated with 7 of 9 unpleasant clusters: Sympathetic Activation, Generalized Pain, GI Distress, Kinesthetic Disconnect, Low Energy, Neurological Discomfort, and Metabolic Stress (*r* = −0.26 - −0.40). In addition, there was a significant but weak inverse correlation between Activated & Attractive and Low Energy (*r* = −0.22) and a significant but weak positive correlation between Physical Intimacy and Heat & Urgency Response (*r* = 0.25).

**Table 4:** Correlations between pleasant and unpleasant factors.

	Physical Alignment	Physical Intimacy	Activated & Attractive	Parasympathetic Activation
**Sympathetic Activation**	−0.06 208	0.03 202	−0.09 202	−0.40** 225
**Generalized Pain**	−0.12 225	0.03 224	−0.07 227	−0.26** 246
**GI Distress**	−0.07 232	0 224	−0.04 227	−0.28** 250
**Illness**	0.07 230	0.07 226	−0.04 229	−0.13 252
**Kinesthetic Disconnect**	−0.16 211	0.03 212	−0.14 213	−0.33** 227
**Low Energy**	−0.14 223	0.01 213	−0.22** 219	−.36** 236
**Neurological Discomfort**	−0.10 221	0.11 217	−0.09 220	−0.30** 242
**Metabolic Stress**	−0.11 210	−0.06 208	−0.05 209	−0.32** 228
**Heat & Urgency Response**	0.06 200	0.25** 197	0.12 201	0.02 213

*Note:* Each cell contains the Pearson *r* value and *n* beneath it. GI = gastrointestinal. **p < .001. Benjamini and Hochberg (1995) corrected significance level = 0.012.

### Initial description of latent factors

Descriptive statistics of the final items in the unpleasant and pleasant latent factors can be found in Table 5. The exploratory *t*-test of global scores found global pleasant scores (*M* = 2.1, *SD* = 0.5) were higher than global unpleasant scores (*M* = 1.4, *SD* = 0.5), indicating pleasant items, on average, occurred more frequently than unpleasant items, *t*(273) = −18.3, *p* < 0.0001. All factors had complete cases from at least 75% of the sample.

**Table 5:** Descriptives of the final unpleasant and pleasant factors from CFA.

		# of items	Complete Cases *% (n)*	Mean Frequency Score *(M* ± *SD)*	Range	Mean Valence Ratings *(M* ± *SD)*
**Unpleasant**	**Sympathetic Activation**	7	97.3 (292)	1.2 ± 0.6	0–3.6	−1.2 ± 0.6
	**Generalized Pain**	6	97.0 (291)	1.5 ± 0.6	0–3.7	−1.6 ± 0.6
	**GI Distress**	4	97.3 (292)	1.4 ± 0.6	0–3.5	−1.8 ± 0.7
	**Illness**	5	97.0 (291)	1.2 ± 0.6	0–3.2	−1.7 ± 0.7
	**Kinesthetic Disconnect**	7	99.0 (297)	1.5 ± 0.6	0–3.7	−1.4 ± 0.6
	**Low Energy**	6	97.3 (292)	2.0 ± 0.6	0–3.8	−1.5 ± 0.6
	**Neurological Discomfort**	4	93.7 (281)	1.0 ± 0.7	0–4.0	−1.2 ± 0.7
	**Metabolic Stress**	5	94.7 (284)	1.0 ± 0.6	0–2.8	−1.4 ± 0.7
	**Heat & Urgency Response**	5	98.3 (295)	1.3 ± 0.6	0–3.0	−1.0 ± 0.6
	**Global Unpleasant**	49	91.3 (274)	1.4 ± 0.5*	0–2.9	−1.4 ± 0.4
**Pleasant**	**Physical Alignment**	5	95.3 (286)	2.1 ± 0.7	0–4.0	1.4 ± 0.8
	**Physical Intimacy**	4	97.0 (291)	1.9 ± 0.8	0–4.0	0.9 ± 0.9
	**Activated & Attractive**	7	97.0 (291)	2.1 ± 0.6	0.4–3.6	1.8 ± 0.7
	**Parasympathetic Activation**	8	99.0 (297)	2.4 ± 0.6	0.5–3.9	2.0 ± 0.7
	**Global Pleasant**	24	93.3 (280)	2.1 ± 0.5*	0.6–3.4	1.5 ± 0.6

*Note:* Global unpleasant and pleasant scores were compared via paired t-test; *p < 0.05.

### Convergent validity and proof-of-concept

**Fig 2:**
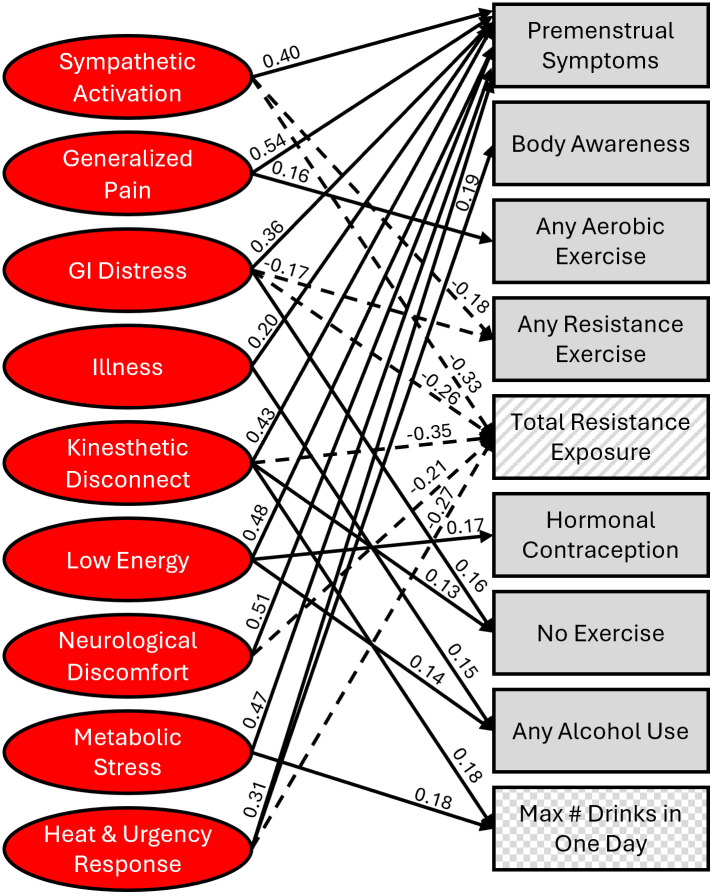
Association of the unpleasant latent factors identified from the CFA (red circles) with scores from validated surveys and lifestyle behavior items (gray rectangles). Each survey and lifestyle behavior was tested *separately*; significant associations from all models are condensed into a single figure to support proof-of-concept for factors. Solid lines denote positive associations; dashed lines denote negative associations. Patterned gray rectangles denote models tested only on a subset of participants (i.e., those who exercised or those who drank at all). Standardized estimates are presented for each significant association.

**Fig 3:**
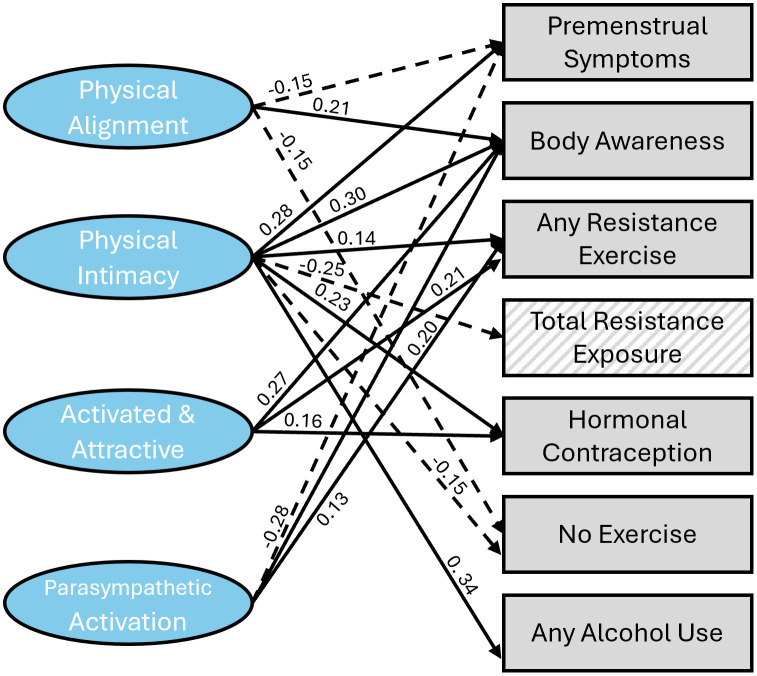
Association of the pleasant latent factors identified from the CFA (blue circles) with scores from validated surveys and lifestyle behavior items (gray rectangles). Each survey and lifestyle behavior was tested separately; significant associations from all models are condensed into a single figure to support proof of concept for factors. Solid lines denote positive relationships; dashed lines denote negative relationships. Patterned gray rectangles denote models tested only on a subset of participants (i.e., those who exercised or those who drank at all). Standardized estimates are presented for each significant association.

To assess convergent validity, the latent factors identified from CFA were associated with existing, conceptually relevant surveys using SEM. Figs 2 and 3 show all significant associations (see S1 Appendix, Tables 4 & 5 for full statistics). We hypothesized that unpleasant latent factors would be positively associated with premenstrual symptoms and negatively associated with body awareness; the opposite relationships were hypothesized for pleasant latent factors. As hypothesized, premenstrual symptom severity from Section 1 of the PSST was positively associated with all nine unpleasant latent factors (Fig 2) and negatively associated with three of the four pleasant latent factors (Physical Alignment, Physical Intimacy, and Parasympathetic Activation; Fig 3). In partial support for the hypotheses, BAQ scores were positively associated with Heat & Urgency Response (Fig 2) and all four pleasant factors (Fig 3).

As proof-of-concept, we expected pleasant latent factors to be positively associated with healthy behaviors (e.g., physically active, little-to-no alcohol consumption) and unpleasant latent factors to be positively associated with unhealthy behaviors (e.g., no exercise, moderate-to-high alcohol consumption). Consistent with these hypotheses, non-participation in exercise was positively associated with GI Distress and Kinesthetic Disconnect (Fig 2) and negatively associated with Physical Intimacy (Fig 3). Consuming any alcohol in the past 30 days was positively associated with Illness and Low Energy (Fig 2); interestingly, it was also positively associated with Physical Intimacy (Fig 3). A higher maximum number of drinks consumed in one drinking day (among drinkers only) was positively associated with Kinesthetic Disconnect and Metabolic Stress (Fig 2).

Participation in any resistance exercise was negatively associated with Sympathetic Activation and GI Distress (Fig 2) and positively associated with Physical Intimacy, Activated & Attractive, and Parasympathetic Activation (Fig 3). Greater resistance training exposure among resistance trained women was negatively associated with Sympathetic Activation, GI Distress, Kinesthetic Disconnect, Neurological Discomfort, and Heat & Urgency Response (Fig 2); however, it was also negatively associated with Physical Intimacy (Fig 3). Participation in aerobic exercise was positively associated with Generalized Pain (Fig 2); no other latent factors were significantly associated with aerobic exercise participation or exposure. Finally, relevant to the main goal of developing a woman-specific somatic inventory, we found that self-reported HC use was positively associated with Low Energy (Fig 2), Physical Intimacy, and Activated & Attractive (Fig 3).

## Discussion

This manuscript describes results from a survey that was developed as an initial step in conceptualizing and developing a women-specific somatic state inventory that captures subjective physical experiences across valences, body systems, and domains of state, sensation, and perception. Using data from 300 young women and an iterative, data- and theory-driven process, this study identified nine unpleasant factors (Kinesthetic Disconnect, Generalized Pain, Gastrointestinal Distress, Illness, Low Energy, Neurological Discomfort, Metabolic Stress, Heat & Urgency Response, and Sympathetic Activation) and four pleasant factors (Physical Alignment, Physical Intimacy, Activated & Attractive, and Parasympathetic Activation) to undergo further testing and validation. This factor structure partially aligned with our hypothesis of four broad-valence factors (kinesthetic, energetic, visceral, wellness) but was far more nuanced: factors were valence-specific and often reflected integrated physiological states. Further, the identified latent factors were associated with relevant validated surveys and lifestyle behaviors to demonstrate proof-of-concept and illustrate the potential research and clinical value of developing such an inventory.

### Somatic experiences can be pleasant or unpleasant

Individuals typically seek treatment and healthcare to alleviate unpleasant sensations, which makes symptom-based assessments prominent in the existing literature. As expected, this made compilation of unpleasant items significantly easier than pleasant items. Expansion of the literature search to include items commonly used in mindfulness techniques, Eastern medicine (e.g., yoga), somatic therapy, and self-reported personal experiences was necessary to gather a sufficient number of pleasant items. Unsurprisingly, there were fewer pleasant items included in the models and fewer pleasant factors retained in the latent factors. Many of the pleasant items were related to physical appearance or more generalized and abstract (e.g., floating, activated, balanced) than unpleasant items, which often referenced specific, commonly experienced physical sensations (e.g., racing heart, headache, dizzy). Despite these challenges, the study achieved a primary goal of identifying four general pleasant domains that can be included in the subsequently developed inventory.

A key observation from assessing pleasant somatic sensations was that global pleasant frequency ratings were higher than global unpleasant frequency ratings. This suggests that pleasant sensations may be perceived as a steady, passive backdrop upon which salient, but perhaps more infrequent, unpleasant sensations punctuate lived experience and draw greater attention. This aligns with the Somatic Marker Hypothesis [1]; a static pleasant physical sensation may be a signal of a system/organism being at homeostasis or functioning at an optimal level. As a result, fewer cognitive and neural resources are allocated to these sensations. On the other hand, unpleasant sensations serve as signals of homeostatic deviation and, when of sufficient intensity, warrant the individual’s attention to correct or alleviate. The stronger a disruptive somatic sensation is, the more attention it draws, cuing decision-making and behavioral action to restore homeostasis [4].

The unpleasant factors were strongly related to one another, as were the pleasant factors. Further research is needed to understand whether such within-valence correlations reflect broader, generalized somatic states (i.e., when one system is out of homeostasis, the person feels “off”) or temperament differences (i.e., some people are oriented towards noticing negative versus positive states). Overall, these results parallel those observed for affect assessment (e.g., Positive and Negative Affect Schedule (PANAS)) [40]. Affect, similar to the somatic items within the present study, appears to consist of two unipolar dimensions, positive and negative, that are weakly related between valences but more strongly within [40,41]. In other words, the experience of unpleasant sensations can be fully distinct from the experience of pleasant sensations. Parasympathetic Activation, however, was the exception: it was inversely associated with seven of the nine unpleasant factors. Items in Parasympathetic Activation relate to a general state of homeostasis and wellness: feeling hydrated, relaxed, rested, comfortable, balanced, and healthy. When reported as occurring more frequently, these items appear to oppose unpleasant sensations. Taken together, these results point to the importance of conceptualizing bodily states as both pleasant and unpleasant, and somatic experiences as both tonic background and momentary experiences.

### Somatic factors often capture integrated physiological states

GI Distress, Neurological Discomfort, and Kinesthetic Disconnect comprised items related to a single body system. Yet, several other unpleasant factors reflected typological clustering of somatic experiences rather than organ/body system-specific categories. Specifically, Sympathetic Activation, Metabolic Stress, Low Energy, and Heat & Urgency Response contained items that spanned more than one body system and instead related to an overall experience that is a composite of each item. To illustrate, Sympathetic Activation items span cardiac, respiratory, and sensory experiences that, together, reflect a common stress state – one that conceptually aligns with psychological states of anxiety and panic. Likewise, Metabolic Stress includes neurological, kinesthetic, and digestive symptoms, all of which parallel common hypoglycemic experiences. Finally, Low Energy items include kinesthetic, energetic, and digestive symptoms related to exhaustion. Thus, the somatic factors are a first step to tapping into higher order patterns of somatic experiences that might better align with both physical and mental health states. This has broad implications for research and clinical assessment of women’s health as it provides a new perspective on defining “well-being” both from an organ-specific, medicalized perspective and an interoceptive, cognitive perspective.

Generalized Pain and Illness appeared to reflect a general physiological domain, but with conceptually related items that may or may not occur concurrently. For example, the first-order cluster of muscle pain, joint pain, and feeling achy reflect musculoskeletal pain, which may or may not co-occur with the first-order cluster of abdominal cramps and lower abdominal pain that reflect digestive or menstrual discomfort. Replication with a larger, more heterogeneous sample (i.e., age range, demographics) is needed to determine whether a single Pain factor is optimal for describing how a woman experiences her body or whether multiple factors that differentiate pain subtypes should be explored. Likewise, Kinesthetic Disconnect items were broader in nature, and included symptoms related to proprioception (e.g., unstable), musculoskeletal agitation (e.g., restless, fidgety), and general physical discomfort (e.g., weak, uncomfortable) which may occur independently, or could, in fact, be related to a general state of unpleasant kinesthesia. Unfortunately, the retrospective method used in this study makes determination of within-factor item co-occurrence or independence impossible but suggests a need for future studies.

Similar observations about the typological clustering of items were made within pleasant factors. For example, whether feeling grounded means you also feel flexible (Physical Alignment); having full breasts means heightened sexual interest and enjoyment (Physical Intimacy); having a good hair day means you also feel radiant and resilient (Activated & Attractive); or being clean means you also feel rested and relaxed (Parasympathetic Activation) is unknown. Yet, it is possible that these somatic experiences share fundamental features that embody a particular state of being. Considerable work remains to further shape and hone the factors identified here and more fully capture how women experience their bodies. Still, the present study clearly illustrates the need to assess pleasant bodily sensations and experiences in women, especially related to healthy physiological sensations.

### Somatic factors are conceptually linked to health and behavior

A primary goal of this study was to initiate the development of an instrument for eventual tracking of comprehensive somatic changes in women across reproductive life stages, in relation to health behaviors, and in response to mental and physical health interventions (e.g., tracking efficacy of substance use or exercise interventions). Thus, exploratory structural equation models were conducted to relate unpleasant and pleasant latent factors to women’s health, somatic, and behavior variables to provide proof-of-concept for the inventory.

The observed relationships of several unpleasant and pleasant latent factors with premenstrual symptoms provide initial validation for the preliminary survey items and the importance of considering somatic state in women’s health. Women who reported greater premenstrual symptoms also broadly reported more frequent unpleasant somatic experiences and less frequent pleasant sensations. This finding parallels prior findings that women with clinical-level menstrual symptoms report greater health anxiety and lower health-related quality of life [9] and may experience premenstrual exacerbation of existing physical and mental conditions (e.g., depression, anxiety, asthma, diabetes, migraines, and irritable bowel syndrome) [42,43]. Importantly, the present somatic items moved beyond classic negative menstrual symptom items to assess a wide range of body systems across a full spectrum of valence. This will allow greater precision in the assessment of individual differences in women’s somatic experiences, particularly in clinical samples where treatment may not only target a reduction in unpleasant sensations, but also an increase in pleasant sensations.

In a separate proof-of-concept analysis, pleasant and unpleasant latent factors were also associated with the use of hormonal contraceptives that included exogenous estradiol and/or progesterone. HC use was positively associated with Physical Intimacy, Activated & Attractive, and Low Energy, potentially implying more frequent experiences of these states among HC users. It is noteworthy that all participants in the HC and NC groups reported 25–35 day cycles; thus, this observation is not related to the absence of menstrual bleeding. Due to the nature of the study, it is not possible to determine whether these relationships were due to neuroendocrine effects of HC or differences in trait-like features related to HC use (e.g., sexual attitudes); however, women who used HC in this study were more likely to be White (versus Asian) and to have recently consumed alcohol compared to naturally cycling women, possibly alluding to mediating socio-cultural and/or behavioral effects. Further, HC use may make women feel safer engaging in sexual activity, thereby increasing the frequency of attracting and engaging in sexual activity [44]. Future research is needed to disentangle neuroendocrine effects from other effects. Moreover, additional research should investigate the utility of measuring somatic experiences in women of different ages and reproductive phases. For example, Heat & Urgency Response has the potential to detect hot flashes in perimenopausal women, with many other possible items of relevance to menarche and pregnancy.

As hypothesized, body awareness was positively associated with pleasant somatic states, supporting prior findings that body awareness positively contributes to physical health and well-being [16–18]. The items that comprise the Body Awareness Questionnaire (BAQ) gauge attentiveness to general physiological states and expectations from those states with vague valences that can be construed as either positively or negatively. Yet, all pleasant factors were associated with total scores from the BAQ. This further aligns our results with the Somatic Marker Theory, interoception, and allostasis theories, each of which predict that unpleasant sensations require less body awareness or interoceptive capacity to register in conscious experience compared to pleasant sensations [1,2,45,46]. The further development of the Women’s Somatic Experiences Inventory may also inform the current controversy regarding validity of many existing interoceptive measures [47–49], and embed women-specific topics in the nascent field of interoception research.

Exercise demonstrated any-versus-none and type-specific effects on somatic states. Not exercising had a dually negative effect. Conceptually, not exercising being positively associated with Kinesthetic Disconnect and negatively associated with Physical Alignment makes sense; those who are less active have less frequent encounters with experiences that require agility, flexibility, or strength (i.e., pleasant kinesthetic function) and may be more likely to experience kinesthetic discomfort. The findings that not exercising is positively associated with GI Distress and negatively associated with Physical Intimacy are relevant considering emerging evidence that sedentary behaviors can modify the gut microbiome and sexual functions [50,51]. Resistance, but not aerobic exercise, had a potent positive association with somatic sensations. Any amount of resistance exercise was positively associated with several pleasant and negatively associated with several unpleasant latent factors that covered a range of body systems, alluding to systemic benefits of participation in resistance exercise by young women. Further, among resistance exercisers, greater exposure yielded greater benefits, indicated by lower scores on five of the nine unpleasant factors. Research on the benefits of resistance training in women across the lifespan support widespread positive impacts on body composition, strength, muscle hypertrophy and preservation, cardiovascular health, and bone density [52,53]. These preliminary findings suggest that the further development of the Women’s Somatic Experience Inventory could benefit women’s health initiatives aimed at increasing physical activity and reducing sedentary behavior in women, especially as they age and become more susceptible to muscle and bone loss.

Finally, alcohol use had fewer associations with somatic factors than might be expected, possibly because the present sample was overtly healthy and mainly reported low-risk use patterns (i.e., 1 day per week, 2–4 drinks per drinking episode). The significant associations that were observed generally support prior studies that have linked immune function [54,55], sleep [56], and sexual activity [57] to alcohol consumption, but neither frequency nor quantity of consumption had any relation with the unpleasant or pleasant somatic factors. Interestingly, greater past 30-day maximal quantity in a single drinking episode was positively associated with Kinesthetic Disconnect and Metabolic Stress, but these factors included items that conceptually align with somatic experiences of intoxication and hangover: spinning, unstable, numb, shaky, weak, hunger pang.

### Limitations

Because of the study’s exploratory nature, results should be interpreted in light of several limitations. Participants were college women, which limits generalizability to other samples of women, particularly considering college students often have a distinct set of health behaviors than women in other life stages. The present study was performed using the initial list of 300 somatic items and an iterative process that combined conceptual and theory-driven searches and quantitative clustering and path modeling techniques to create the preliminary factor structure of 73 items. The original item list of 300 was refined using pre-data and post-data conceptual steps, a data-driven step, and a post hoc conceptual step. Multiple imputation was performed with a Missing at Random assumption, but this was not explicitly tested. The CFI and TLI values for the CFA models for the unpleasant factors (and TLI for the pleasant factors) were below the range typically considered acceptable. Thus, results should be replicated in other samples and undergo additional rigorous psychometric and validity testing. However, considering the preliminary nature and iterative strategies used of inventory development, we propose that the pilot inventory (S2 Appendix File) may be a useful starting point for further study. For SEM models that evaluated associations between latent factors and within-behaviors (i.e., within drinkers or within exercisers), sample sizes were reduced and smaller than recommended, which may have inflated the estimates. Relevant confounding factors such as sexual activity, sexual orientation, religion, and other sociocultural factors were not assessed in this sample but may contribute to both the perception and interoceptive interpretation of somatic experiences. Moreover, comprehensive assessment of mental health concerns and unhealthy lifestyle behaviors were not fully assessed; these factors may affect the generalizability of the factors that were identified. Future research may wish to consider certain dropped somatic items that may be more pertinent to other life or reproductive stages. The names given to the identified factors may require additional refinement (e.g., Heat & Urgency Responses) and/or validation with physiological data (e.g., Parasympathetic Activation). Missing data were present across all factors due to participant’s ability to mark items as “Unclear” and non-requirement of full survey completion. Finally, the survey design consisted of a cross-sectional assessment of retrospective experiences; this introduced the potential for memory biases to skew the data. Nonetheless, this study provides a strong path forward to improve understanding of women’s subjective experiences of their bodies and uncover how these experiences relate to behavior and overall health.

## Conclusions

This is the first study to conceptualize and build a comprehensive factor structure of women’s somatic states, bodily experiences, and physical perceptions that covers multiple body systems and includes both pleasant and unpleasant sensations. Findings provide novel insights into the subjective somatic experiences of young women and their relationship to menstrual cycle factors (premenstrual symptoms and hormonal contraception use), body awareness, and lifestyle behaviors (exercise and alcohol use). Important next steps would be to translate the final list of 49 unpleasant and 24 pleasant items into practical subscale scores for applied use, optimize survey flow, perform comprehensive psychometric validation, and test the inventory in samples of women that vary in age, reproductive phase, physical health, and lifestyles. By doing so, it should be possible to create a Women’s Somatic Experience Inventory that has the potential to expand current conceptualization of women’s health – tapping into links between physical and psychological well-being, the experience of and response to momentary stress states, and interoceptive factors that influence clinical outcomes related to menarche, pregnancy, menopause, and other female-specific concerns.

## Supporting information

S1 Appendix Full list of somatic items used in survey and supplemental tables. (DOCX)

S2 Appendix Women’s Somatic Experiences Inventory: Pilot Form. (DOCX)

## References

[1] Damasio AR. The somatic marker hypothesis and the possible functions of the prefrontal cortex. Philos Trans R Soc Lond B Biol Sci. 1996;351(1346):1413–20. doi: 10.1098/rstb.1996.0125 8941953

[2] Chen WG, Schloesser D, Arensdorf AM, Simmons JM, Cui C, Valentino R, et al. The Emerging Science of Interoception: Sensing, Integrating, Interpreting, and Regulating Signals within the Self. Trends Neurosci. 2021;44(1):3–16. doi: 10.1016/j.tins.2020.10.007 33378655 PMC7780231

[3] Khalsa SS, Lapidus RC. Can interoception improve the pragmatic search for biomarkers in psychiatry?. Front Psychiatry. 2016;7. doi: 10.3389/fpsyt.2016.00121 PMC4958623 27504098

[4] Damasio A, Carvalho GB. The nature of feelings: evolutionary and neurobiological origins. Nat Rev Neurosci. 2013;14(2):143–52. doi: 10.1038/nrn3403 23329161

[5] Critchley HD, Harrison NA. Visceral influences on brain and behavior. Neuron. 2013;77(4):624–38. doi: 10.1016/j.neuron.2013.02.008 23439117

[6] Romans S, Clarkson R, Einstein G, Petrovic M, Stewart D. Mood and the menstrual cycle: a review of prospective data studies. Gend Med. 2012;9(5):361–84. doi: 10.1016/j.genm.2012.07.003 23036262

[7] Schmalenberger KM, Tauseef HA, Barone JC, Owens SA, Lieberman L, Jarczok MN, et al. How to study the menstrual cycle: Practical tools and recommendations. Psychoneuroendocrinology. 2021;123:104895. doi: 10.1016/j.psyneuen.2020.104895 33113391 PMC8363181

[8] Bertone-Johnson ER, Hankinson SE, Johnson SR, Manson JE. Timing of alcohol use and the incidence of premenstrual syndrome and probable premenstrual dysphoric disorder. J Womens Health (Larchmt). 2009;18(12):1945–53. doi: 10.1089/jwh.2009.1468 20044856 PMC2828255

[9] Craner J, Sigmon S, Martinson A, McGillicuddy M. Perceptions of health and somatic sensations in women reporting premenstrual syndrome and premenstrual dysphoric disorder. J Nerv Ment Dis. 2013;201(9):780–5. doi: 10.1097/NMD.0b013e3182a213f1 23995034

[10] Kaiser G, Janda C, Kleinstäuber M, Weise C. Clusters of premenstrual symptoms in women with PMDD: Appearance, stability and association with impairment. J Psychosom Res. 2018;115:38–43. doi: 10.1016/j.jpsychores.2018.10.004 30470315

[11] Brown SG, Morrison LA, Larkspur LM, Marsh AL, Nicolaisen N. Well-being, sleep, exercise patterns, and the menstrual cycle: a comparison of natural hormones, oral contraceptives and depo-provera. Women Health. 2008;47(1):105–21. doi: 10.1300/J013v47n01_06 18581695

[12] Logue CM, Moos RH. Positive perimenstrual changes: toward a new perspective on the menstrual cycle. J Psychosom Res. 1988;32(1):31–40. doi: 10.1016/0022-3999(88)90086-4 3042993

[13] McPherson ME, Korfine L. Menstruation across time: menarche, menstrual attitudes, experiences, and behaviors. Womens Health Issues. 2004;14(6):193–200. doi: 10.1016/j.whi.2004.08.006 15589769

[14] Stewart DE. Positive changes in the premenstrual period. Acta Psychiatr Scand. 1989;79(4):400–5. doi: 10.1111/j.1600-0447.1989.tb10276.x 2735211

[15] Shields SA, Mallory ME, Simon A. The Body Awareness Questionnaire: Reliability and Validity. Journal of Personality Assessment. 1989;53(4):802–15. doi: 10.1207/s15327752jpa5304_16

[16] Açik M, Çağiran Yilmaz F. Body awareness mediates the relationship between body mass index and lipid profiles in adolescents. J Diabetes Metab Disord. 2022;21(1):589–97. doi: 10.1007/s40200-022-01021-3 35673458 PMC9167376

[17] Kalkışım ŞN, Çan MA, Erden A, Uzun Ö, Ertemoğlu Öksüz C, Zihni NB. Relationships between anthropometric measurements, muscle strength and body awareness. Acta Neurol Belg. 2022;122(1):31–42. doi: 10.1007/s13760-020-01578-x 33661514

[18] Kalkışım ŞN, Erden A, Kanber Uzun Ö, Ertemoğlu Öksüz C, Zihni NB, Çan MA. Relationship between body awareness level and musculoskeletal pain complaints, physical activity level and emotional status in healthy people. Acta Neurol Belg. 2023;123(5):1789–96. doi: 10.1007/s13760-022-02056-2 35947302

[19] Karakus A, Inanc S, Akyurek G. Neurocognitive function, psychosocial characteristics, and occupational performance across menstrual phases in young adults with and without primary dysmenorrhea. Eur J Obstet Gynecol Reprod Biol. 2026;318:114965. doi: 10.1016/j.ejogrb.2026.114965 41547324

[20] Kovács Z, Atombosiye E, Hegyi G, Szőke H. The Effect of Aviva Exercise Intervention on Pain Level and Body Awareness in Women with Primary Dysmenorrhea. Medicina (Kaunas). 2024;60(1):184. doi: 10.3390/medicina60010184 38276063 PMC10821191

[21] Kiloatar H, Kurt G. Perception of benefits-barriers of exercise, physical activity level, and body awareness in women with premenstrual syndrome. J Obstet Gynaecol Res. 2024;50(1):120–7. doi: 10.1111/jog.15822 37919793

[22] Günebakan Ö, Acar M. The effect of tele-yoga training in healthy women on menstrual symptoms, quality of life, anxiety-depression level, body awareness, and self-esteem during COVID-19 pandemic. Ir J Med Sci. 2023;192(1):467–79. doi: 10.1007/s11845-022-02985-0 35332504 PMC8945871

[23] Karaaslan Y, Ucuzoglu ME, Yüksel S, Yılmaz Yalçınkaya E. The relationship of pain, disability, physical activity, and body awareness with kinesiophobia in pregnant women with low back pain. Somatosens Mot Res. 2023;40(4):156–60. doi: 10.1080/08990220.2023.2263547 37787051

[24] Turan A, Gerçek H, Tüzmen HD, Horasanli JE. Investigation of predictors of body awareness of pregnant women. Rev Assoc Med Bras (1992). 2025;71(6):e20241931. doi: 10.1590/1806-9282.20241931 40638463 PMC12245064

[25] Gözgen H, Belgen Kaygısız B. Effectiveness of body awareness therapy on pain and pain coping strategies in postmenopausal women: A randomized controlled study. J Back Musculoskelet Rehabil. 2025;38(6):1418–28. doi: 10.1177/10538127251339819 40350621

[26] Sahin G, Pekyavas NO, Aytar A. Relaxation training for vasomotor symptoms and body awareness in menopausal and postmenopausal women. J Bodyw Mov Ther. 2025;45:502–7. doi: 10.1016/j.jbmt.2025.09.021 41316612

[27] Farage MA, Neill S, MacLean AB. Physiological changes associated with the menstrual cycle: a review. Obstet Gynecol Surv. 2009;64(1):58–72. doi: 10.1097/OGX.0b013e3181932a37 19099613

[28] Farage MA, Osborn TW, MacLean AB. Cognitive, sensory, and emotional changes associated with the menstrual cycle: a review. Arch Gynecol Obstet. 2008;278(4):299–307. doi: 10.1007/s00404-008-0708-2 18592262

[29] Steiner M, Macdougall M, Brown E. The premenstrual symptoms screening tool (PSST) for clinicians. Arch Womens Ment Health. 2003;6(3):203–9. doi: 10.1007/s00737-003-0018-4 12920618

[30] Eisenlohr-Moul TA. Commentary on Joyce *et al*.: Studying menstrual cycle effects on behavior requires within-person designs and attention to individual differences in hormone sensitivity. Addiction. 2021;116(10):2759–60. doi: 10.1111/add.15576 34048110 PMC8429129

[31] Kiesner J. One woman’s low is another woman’s high: Paradoxical effects of the menstrual cycle. Psychoneuroendocrinology. 2011;36(1):68–76. doi: 10.1016/j.psyneuen.2010.06.007 20650571

[32] Kiesner J, Pastore M. Day-to-day co-variations of psychological and physical symptoms of the menstrual cycle: insights to individual differences in steroid reactivity. Psychoneuroendocrinology. 2010;35(3):350–63. doi: 10.1016/j.psyneuen.2009.07.011 19729249

[33] Sobell LC, Sobell MB. Timeline Follow-Back. Measuring Alcohol Consumption. Totowa, NJ: Humana Press. 1992. p. 41–72. doi: 10.1007/978-1-4612-0357-5_3

[34] Mehling WE, Price C, Daubenmier JJ, Acree M, Bartmess E, Stewart A. The Multidimensional Assessment of Interoceptive Awareness (MAIA). PLoS One. 2012;7(11):e48230. doi: 10.1371/journal.pone.0048230 23133619 PMC3486814

[35] Mehling WE, Acree M, Stewart A, Silas J, Jones A. The Multidimensional Assessment of Interoceptive Awareness, Version 2 (MAIA-2). PLoS One. 2018;13(12):e0208034. doi: 10.1371/journal.pone.0208034 30513087 PMC6279042

[36] Muthen LK, Muthen BO. Mplus User’s Guide. 8th ed. Los Angeles, CA: Muthen & Muthen. 1998.

[37] Hu L, Bentler PM. Cutoff criteria for fit indexes in covariance structure analysis: Conventional criteria versus new alternatives. Structural Equation Modeling: A Multidisciplinary Journal. 1999;6(1):1–55. doi: 10.1080/10705519909540118

[38] Xia Y, Yang Y. RMSEA, CFI, and TLI in structural equation modeling with ordered categorical data: The story they tell depends on the estimation methods. Behav Res Methods. 2019;51(1):409–28. doi: 10.3758/s13428-018-1055-2 29869222

[39] Hooper D, Coughlan J, Mullen MR. Structural equation modelling: Guidelines for determining model fit. Electronic J Business Research Methods. 2008;6:53–60.

[40] Diener E, Emmons RA. The independence of positive and negative affect. J Pers Soc Psychol. 1984;47(5):1105–17. doi: 10.1037//0022-3514.47.5.1105 6520704

[41] Zevon MA, Tellegen A. The structure of mood change: An idiographic/nomothetic analysis. J Personality and Social Psychology. 1982;43(1):111–22. doi: 10.1037/0022-3514.43.1.111

[42] Pinkerton JV, Guico-Pabia CJ, Taylor HS. Menstrual cycle-related exacerbation of disease. Am J Obstet Gynecol. 2010;202(3):221–31. doi: 10.1016/j.ajog.2009.07.061 20207238 PMC3107848

[43] Kiesner J, Mendle J, Eisenlohr-Moul TA, Pastore M. Cyclical Symptom Change Across the Menstrual Cycle. Clinical Psychological Science. 2016;4(5):882–94. doi: 10.1177/2167702616635031

[44] Both S, Lew-Starowicz M, Luria M, Sartorius G, Maseroli E, Tripodi F, et al. Hormonal contraception and female sexuality: position statements from the European Society of Sexual Medicine (ESSM). The Journal of Sexual Medicine. 2019;16:1681–95. doi: 10.1016/j.jsxm.2019.08.005 31521571

[45] McEwen BS. Stress, adaptation, and disease. Allostasis and allostatic load. Ann N Y Acad Sci. 1998;840:33–44. doi: 10.1111/j.1749-6632.1998.tb09546.x 9629234

[46] Sterling P. Allostasis: A model of predictive regulation. Physiology & Behavior. 2012;106:5–15. doi: 10.1016/j.physbeh.2011.06.004 21684297

[47] Desmedt O, Heeren A, Corneille O, Luminet O. What do measures of self-report interoception measure? Insights from a systematic review, latent factor analysis, and network approach. Biol Psychol. 2022;169:108289. doi: 10.1016/j.biopsycho.2022.108289 35150768

[48] Murphy J, Catmur C, Bird G. Classifying individual differences in interoception: Implications for the measurement of interoceptive awareness. Psychon Bull Rev. 2019;26(5):1467–71. doi: 10.3758/s13423-019-01632-7 31270764 PMC6797703

[49] Garfinkel SN, Seth AK, Barrett AB, Suzuki K, Critchley HD. Knowing your own heart: distinguishing interoceptive accuracy from interoceptive awareness. Biol Psychol. 2015;104:65–74. doi: 10.1016/j.biopsycho.2014.11.004 25451381

[50] Bressa C, Bailén-Andrino M, Pérez-Santiago J, González-Soltero R, Pérez M, Montalvo-Lominchar MG, et al. Differences in gut microbiota profile between women with active lifestyle and sedentary women. PLoS One. 2017;12(2):e0171352. doi: 10.1371/journal.pone.0171352 28187199 PMC5302835

[51] Cabral PUL, Canário ACGMD, Spyrides MHC, Uchôa SA da C, Eleutério Júnior J, Giraldo PC, et al. Physical activity and sexual function in middle-aged women. Rev Assoc Med Bras (1992). 2014;60(1):47–52. doi: 10.1590/1806-9282.60.01.011 24918852

[52] Hagstrom AD, Marshall PW, Halaki M, Hackett DA. The Effect of Resistance Training in Women on Dynamic Strength and Muscular Hypertrophy: A Systematic Review with Meta-analysis. Sports Med. 2020;50(6):1075–93. doi: 10.1007/s40279-019-01247-x 31820374

[53] Kraemer WJ, Fragala MS, Ratamess NA. Evolution of resistance training in women: History and mechanisms for health and performance. Sports Med Health Sci. 2025;7(5):351–65. doi: 10.1016/j.smhs.2025.01.005 40936659 PMC12421175

[54] Afshar M, Richards S, Mann D, Cross A, Smith GB, Netzer G, et al. Acute immunomodulatory effects of binge alcohol ingestion. Alcohol. 2015;49(1):57–64. doi: 10.1016/j.alcohol.2014.10.002 25572859 PMC4314366

[55] Molina PE, Happel KI, Zhang P, Kolls JK, Nelson S. Focus on: Alcohol and the immune system. Alcohol Res Health. 2010;33(1–2):97–108. 23579940 PMC3887500

[56] Ebrahim IO, Shapiro CM, Williams AJ, Fenwick PB. Alcohol and sleep I: effects on normal sleep. Alcohol Clin Exp Res. 2013;37(4):539–49. doi: 10.1111/acer.12006 23347102

[57] George WH, Stoner SA. Understanding acute alcohol effects on sexual behavior. Annual Review of Sex Research. 2000;11:92–124. doi: 10.1080/10532528.2000.10559785 11351836

