# Supplementary material for: Capturing women’s bodily experiences: Conception and formative stages of the women’s somatic experience inventory
Source: PLoS One. 2026 Jul 13;21(7):e0353167. doi: 10.1371/journal.pone.0353167 (PMC13362119; doi:10.1371/journal.pone.0353167)
Supplement: S1 Appendix — (DOCX) [file pone.0353167.s001.docx]

**S1 Appendix. Full list of somatic items used in survey, in alphabetical order**

Abdominal cramping

Abdominal pain

Achy

Activated

Agile

Aware of where body is in space

Bad taste in mouth

Balanced

Blemished skin

Bloated

Body rush

Breathless

Bubbly

Burning eyes

Buzzing

Calm breath

Centered

Cheeks warm

Chest pain

Clammy skin

Clean

Clear vision

Clumsy

Clunky

Cold

Cold limbs

Comfortable

Congested

Connected

Constipated

Cool

Coordinated

Cough

Cough

Decreased appetite

Decreased sexual enjoyment

Deep breathing

Deft

Diarrhea

Difficulty swallowing

Dirty

Dirty

Disjointed

Dizzy

Drowsy

Dry eyes

Dry mouth

Dry nose

Dry skin

Embodied

Energetic

Expansive

Face numb

Faint

Fatigued

Feminine

Feverish

Fidgety

Fiery

Flexible

Floating

Fluid

Flushed

Frozen

Full

Full breasts

Full breathing

Gassy

Good hair

Graceful

Grounded

Gurgling

Head empty

Head numb

Head spinning

Headache

Heady

Healthy

Heart palpitations

Heart thudding

Heavy

Heavy head

Heavy limbs

Heightened sexual interest

High

Hot

Hot limbs

Hunger pang

Hungry

Hydrated

Ill

In good health

In your head

Increased appetite

Increased sexual enjoyment

Inflated

Itchy

Jerky

Jittery

Joint pain

Jumpy

Keyed up

Lean

Lightened

Limber

Lips numb

Loose

Lower abdominal pain or cramping

Lowered body pain

Lubricated

Lump in stomach

Lump in throat

Mobile

Muscle pain

Nauseated

Nauseous

Nimble

Not bloated

Numb

Numb all over

Oily skin

Out of touch

Overheated

Overstimulated

Painful

Palms sweaty

Pelvic pain or pressure

Physically attractive

Physically unattractive

Pins and needles

Powerful

Puffy

Radiant

Rapid breathing

Relaxed

Relaxed

Relaxed muscles

Resilient

Responsive

Rested

Restless

Runny nose

Satiated

Sedated

Sexy

Shaky

Shallow breathing

Sick

Sleepy

Slouchy

Slow

Slow heart beat

Sluggish

Smooth breath

Smooth digestion

Smooth skin

Sore throat

Spinning

Sprightly

Stable

Stacked

Stiff

Stomach bloated

Stomach growling

Stomach tightening

Strong

Strong smell

Stuffy nose

Sudden thirst pangs

Sudden urge to defecate

Sudden urge to urinate

Supple

Supported

Sweaty

Swirling

Tender breasts

Tense

Thick tongue

Thirsty

Tickle in throat

Tingly

Tingly

Trapped

Trouble swallowing

Tunnel vision

Twitchy

Ugly

Uncomfortable

Unhygienic

Unstable

Upset stomach

Vibrating

Vigorous

Vigorous

Wanting physical contact

Warm

Warm limbs

Watery or teary eyes

Weak

Well

Well groomed

**S1 Table 1. Correlations among unpleasant clusters (from PROC VARCLUS) frequency sum scores.**

|  | **Sympathetic Activation** | **Generalized Pain** | **GI Distress** | **Illness** | **Kinesthetic Disconnect** | **Low Energy** | **Neurological Discomfort** | **Metabolic Stress** | **Heat & Urgency Response** |
| --- | --- | --- | --- | --- | --- | --- | --- | --- | --- |
| **Sympathetic Activation** | —  238 |  |  |  |  |  |  |  |  |
| **Generalized Pain** | **0.51****  230 | —  269 |  |  |  |  |  |  |  |
| **GI Distress** | **0.44****  235 | **0.47****  265 | —  273 |  |  |  |  |  |  |
| **Illness** | **0.32****  236 | **0.31****  267 | **0.25****  270 | —  275 |  |  |  |  |  |
| **Kinesthetic Disconnect** | **0.67****  214 | **0.51****  237 | **0.52****  239 | **0.29****  241 | —  241 |  |  |  |  |
| **Low Energy** | **0.61****  223 | **0.54****  251 | **0.46****  255 | **0.34****  254 | **0.68****  230 | —  257 |  |  |  |
| **Neurological Discomfort** | **0.63****  225 | **0.56****  254 | **0.36****  256 | **0.41****  260 | **0.63****  231 | **0.57****  242 | —  266 |  |  |
| **Metabolic Stress** | **0.65****  219 | **0.60****  219 | **0.46****  241 | **0.26****  240 | **0.59****  220 | **0.58****  228 | **0.54****  232 | —  249 |  |
| **Heat & Urgency Response** | **0.43****  203 | **0.37****  225 | **0.42****  227 | **0.26****  240 | **0.48****  210 | **0.37****  216 | **0.47****  219 | **0.44****  208 | —  229 |

*Note:* Each cell contains the Pearson *r* value and *n* beneath it. GI = gastrointestinal. ***p*<.001; Benjamini and Hochberg (1995) corrected significance level = 0.05.

**S1 Table 2. Model fit indices for Confirmatory Factor Analyses.**

|  | **AIC** | **BIC** | **RMSEA** | **CFI** | **TLI** | **SRMR** |
| --- | --- | --- | --- | --- | --- | --- |
| **Unpleasant** | 32340.2 | 33016.1 | 0.046 | 0.85 | 0.84 | 0.056 |
| **Pleasant** | 15736.3 | 16024.4 | 0.048 | 0.90 | 0.89 | 0.059 |

*Note:* AIC = Akaike Information Criteria; BIC = Bayesian Information Criteria; RMSEA = Root Mean Square Error of Approximation; CFI = Comparative Fit Index; TLI = Tucker-Lewis Index; SRMR = Standard Root Mean Square Residual

**S1 Table 3. Correlations among pleasant clusters (from PROC VARCLUS) frequency sum scores.**

|  | **Physical Alignment** | **Physical Intimacy** | **Activated & Attractive** | **Parasympathetic Activation** |
| --- | --- | --- | --- | --- |
| **Physical Alignment** | —  240 |  |  |  |
| **Physical Intimacy** | **0.51****  200 | —  226 |  |  |
| **Activated & Attractive** | **0.51****  203 | **0.40****  200 | —  230 |  |
| **Parasympathetic Activation** | **0.63****  218 | **0.42****  216 | **0.64****  217 | —  252 |

*Note:* Each cell contains the Pearson *r* value and *n* beneath it. ***p*<.001; Benjamini and

Hochberg (1995) corrected significance level = 0.05.

**S1 Table 4.** **Results from independent Structural Equation Models associating menstrual cycle factors, body awareness, and health behaviors (rows) to the identified unpleasant somatic factors (columns).**

|  | Sympathetic Activation | Generalized Pain | GI Distress | Illness | Kinesthetic Disconnect | Low Energy | Neurological Discomfort | Metabolic Stress | Heat & Urgency Response |
| --- | --- | --- | --- | --- | --- | --- | --- | --- | --- |
| Premenstrual symptoms | **0.40 (0.06)** | **0.54 (0.05)** | **0.36 (0.07)** | **0.2 (0.06)** | **0.43 (0.06)** | **0.48 (0.05)** | **0.51 (0.05)** | **0.47 (0.06)** | **0.32 (0.07)** |
| Body awareness | -0.005 (0.07) | 0.12 (0.07) | 0.02 (0.07) | -0.06 (0.07) | -0.04 (0.07) | 0.07 (0.07) | -0.01 (0.07) | 0.11 (0.07) | **0.19** (0.07) |
| Hormonal contraception | 0.02 (0.06) | 0.12 (0.07) | 0.13 (0.07) | 0.06 (0.07) | 0.08 (0.06) | **0.17 (0.06)** | 0.001 (0.07) | 0.02 (0.07) | 0.05 (0.07) |
| No exercise | 0.06 (0.07) | -0.09 (0.07) | **0.16 (0.07)** | 0.06 (0.07) | **0.13 (0.06)** | 0.08 (0.07) | -0.008 (0.07) | 0.01 (0.07) | 0.02 (0.07) |
| Any aerobic exercise | 0.04 (0.06) | **0.16 (0.07)** | 0.02 (0.07) | -0.02 (0.07) | -0.01 (0.06) | 0.02 (0.07) | 0.07 (0.07) | 0.09 (0.07) | 0.04 (0.07) |
| Any resistance exercise | **-0.18 (0.08)** | -0.05 (0.08) | **-0.17 (0.08)** | -0.09 (0.08) | -0.12 (0.08) | -0.09 (0.08) | -0.1 (0.10) | -0.1 (0.08) | -0.07 (0.07) |
| Aerobic exercise quantity | -0.08 (0.07) | 0.11 (0.08) | -0.01 (0.08) | 0.09 (0.08) | -0.01 (0.08) | 0.03 (0.08) | 0.03 (0.08) | 0.02 (0.08) | -0.04 (0.09) |
| Resistance exercise quantity | **-0.33 (0.10)** | -0.19 (0.11) | **-0.26 (0.12)** | -0.14 (0.12) | **-0.35 (0.10)** | -0.12 (0.11) | **-0.21 (0.10)** | -0.1 (0.12) | **-0.27 (0.10)** |
| Drinking status | 0.11 (0.06) | 0.13 (0.07) | 0.1 (0.07) | **0.15 (0.06)** | 0.12 (0.06) | **0.14 (0.06)** | 0.09 (0.07) | 0.09 (0.07) | 0.07 (0.07) |
| Drinking frequency | 0.04 (0.09) | 0.14 (0.09) | 0.17 (0.10) | 0.03 (0.09) | 0.08 (0.09) | 0.02 (0.09) | 0.13 (0.09) | 0.16 (0.09) | 0.14 (0.09) |
| Drinking quantity | -0.02 (0.09) | 0.02 (0.09) | 0.08 (0.10) | 0.04 (0.09) | 0.05 (0.09) | 0.03 (0.09) | 0.01 (0.09) | 0.16 (0.09) | 0.05 (0.10) |
| Max drink quantity | 0.09 (0.09) | 0.11 (0.09) | 0.08 (0.10) | 0.11 (0.09) | **0.18 (0.08)** | 0.07 (0.09) | 0.14 (0.08) | **0.18 (0.09)** | 0.07 (0.09) |

*Note:* Data presented as standardized coefficient (standard error). Values in bold are significant at *p* < .05.

**S1 Table 5.** **Results from independent Structural Equation Models associating menstrual cycle factors, body awareness, and health behaviors (rows) to the identified pleasant somatic factors (columns).**

|  | Physical Alignment | Physical Intimacy | Activated & Attractive | Parasympathetic Activation |
| --- | --- | --- | --- | --- |
| Premenstrual symptoms | **-0.15 (0.07)** | **0.28 (0.06)** | -0.04 (0.07) | **-0.28 (0.06)** |
| Body awareness | **0.21 (0.07)** | **0.30 (0.06)** | **0.27 (0.06)** | **0.20 (0.06)** |
| Hormonal contraception | 0.12 (0.07) | **0.23 (0.06)** | **0.16 (0.07)** | -0.02 (0.06) |
| No exercise | **-0.15 (0.07)** | **-0.15 (0.07)** | -0.04 (0.07) | -0.12 (0.06) |
| Any aerobic exercise | 0.01 (0.07) | 0.07 (0.07) | -0.02 (0.07) | -0.04 (0.06) |
| Any resistance exercise | 0.13 (0.07) | **0.14 (0.07)** | **0.21 (0.06)** | **0.13 (0.06)** |
| Aerobic exercise quantity | 0.09 (0.08) | 0.10 (0.08) | -0.01 (0.08) | 0.01 (0.07) |
| Resistance exercise quantity | -0.01 (0.12) | **-0.25 (0.11)** | -0.03 (0.12) | 0.15 (0.11) |
| Drinking status | -0.02 (0.07) | **0.34 (0.06)** | 0.10 (0.07) | -0.03 (0.06) |
| Drinking frequency | -0.13 (0.09) | 0.03 (0.10) | 0.01 (0.09) | -0.05 (0.08) |
| Drinking quantity | -0.11 (0.09) | 0.02 (0.10) | 0.01 (0.09) | -0.05 (0.08) |
| Max drink quantity | -0.1 (0.09) | -0.07 (0.10) | -0.09 (0.09) | -0.09 (0.08) |

*Note:* Data presented as standardized coefficient (standard error). Values in bold are significant at *p* < .05.
