# Supplementary material for: Capturing women’s bodily experiences: Conception and formative stages of the women’s somatic experience inventory
Source: PLoS One. 2026 Jul 13;21(7):e0353167. doi: 10.1371/journal.pone.0353167 (PMC13362119; doi:10.1371/journal.pone.0353167)
Supplement: S2 Appendix — (DOCX) [file pone.0353167.s002.docx]

**S2 File. Women’s Somatic Experiences Inventory: Pilot Form**

****Note: This is a pilot/preliminary version of WSEI; further refinement and validation is necessary prior to use. Item order has been randomized.***

Below is a list of words or phrases that describe physical sensations you may experience on a day-to-day basis.

Indicate how often you experienced each item over the **last 30 days**. Do your best to indicate items you would use to describe your own **personal, physical experience of your body.** There are no right or wrong answers.

If you have not experienced an item in the last 30 days, select “**Never**”.

|  | **Never**  (0) | **Rarely**  (1) | **Sometimes**  (2) | **Most of the time**  (3) | **Always**  (4) |
| --- | --- | --- | --- | --- | --- |
| 1. Shaky |  |  |  |  |  |
| 1. Sore throat |  |  |  |  |  |
| 1. Resilient |  |  |  |  |  |
| 1. Good hair |  |  |  |  |  |
| 1. Numb |  |  |  |  |  |
| 1. Runny nose |  |  |  |  |  |
| 1. Overheated |  |  |  |  |  |
| 1. Wanting physical contact |  |  |  |  |  |
| 1. Dizzy |  |  |  |  |  |
| 1. Tense |  |  |  |  |  |
| 1. Breathless |  |  |  |  |  |
| 1. Radiant |  |  |  |  |  |
| 1. Pins and needles |  |  |  |  |  |
| 1. Rested |  |  |  |  |  |
| 1. Stable |  |  |  |  |  |
| 1. Diarrhea |  |  |  |  |  |
| 1. Bloated |  |  |  |  |  |
| 1. Tender breasts |  |  |  |  |  |
| 1. Physically attractive |  |  |  |  |  |
| 1. Hunger pang |  |  |  |  |  |
| 1. Calm breath |  |  |  |  |  |
| 1. Spinning |  |  |  |  |  |
| 1. Slouchy |  |  |  |  |  |
| 1. Sluggish |  |  |  |  |  |
| 1. Grounded |  |  |  |  |  |
| 1. Constipation |  |  |  |  |  |
| 1. Weak |  |  |  |  |  |
| 1. Unstable |  |  |  |  |  |
| 1. Clean |  |  |  |  |  |
| 1. Dry mouth |  |  |  |  |  |
|  | **Never**  (0) | **Rarely**  (1) | **Sometimes**  (2) | **Most of the time**  (3) | **Always**  (4) |
| 1. Heavy |  |  |  |  |  |
| 1. Healthy |  |  |  |  |  |
| 1. Feverish |  |  |  |  |  |
| 1. Cheeks warm |  |  |  |  |  |
| 1. Upset stomach |  |  |  |  |  |
| 1. Burning in stomach |  |  |  |  |  |
| 1. Agile |  |  |  |  |  |
| 1. Coordinated |  |  |  |  |  |
| 1. Sudden thirst |  |  |  |  |  |
| 1. Racing heart |  |  |  |  |  |
| 1. Full breasts |  |  |  |  |  |
| 1. Fatigued |  |  |  |  |  |
| 1. Warm limbs |  |  |  |  |  |
| 1. Stuffy nose |  |  |  |  |  |
| 1. Sudden urge to urinate |  |  |  |  |  |
| 1. Faint |  |  |  |  |  |
| 1. Nauseous |  |  |  |  |  |
| 1. Comfortable |  |  |  |  |  |
| 1. Flexible |  |  |  |  |  |
| 1. Centered |  |  |  |  |  |
| 1. Gassy |  |  |  |  |  |
| 1. Rapid breath |  |  |  |  |  |
| 1. Energetic |  |  |  |  |  |
| 1. Sick |  |  |  |  |  |
| 1. Balanced |  |  |  |  |  |
| 1. Tunnel vision |  |  |  |  |  |
| 1. Fidgety |  |  |  |  |  |
| 1. Increased sexual enjoyment |  |  |  |  |  |
| 1. Muscle pain |  |  |  |  |  |
| 1. Cough |  |  |  |  |  |
| 1. Tingly |  |  |  |  |  |
| 1. Sudden urge to defecate |  |  |  |  |  |
| 1. Achy |  |  |  |  |  |
| 1. Heightened sexual interest |  |  |  |  |  |
| 1. Relaxed |  |  |  |  |  |
| 1. Lower abdominal pain |  |  |  |  |  |
| 1. Sleepy |  |  |  |  |  |
| 1. Hydrated |  |  |  |  |  |
| 1. Uncomfortable |  |  |  |  |  |
| 1. Headache |  |  |  |  |  |
| 1. Powerful |  |  |  |  |  |
| 1. Head spinning |  |  |  |  |  |
| 1. Decreased sexual enjoyment |  |  |  |  |  |

**Unpleasant Subscale Items**

**Sympathetic Activation:** 11, 30, 39, 40, 46, 52, 56

**Generalized Pain:** 18, 47, 59, 63, 66, 70

**GI Distress:** 16, 26, 35, 51

**Illness:** 2, 6, 44, 54, 60

**Kinesthetic Disconnect:** 5, 10, 27, 28, 57, 69, 73

**Low Energy:** 17, 23, 24, 31, 42, 67

**Neurological Discomfort:** 9, 13, 61, 72

**Metabolic Stress:** 1, 20, 22, 33, 36

**Heat & Urgency Response:** 7, 34, 43, 45, 62

**Pleasant Subscale Items**

**Physical Alignment:** 15, 25, 37, 38, 49

**Physical Intimacy:** 8, 41, 58, 64

**Activated & Attractive:** 3, 4, 12, 19, 50, 53, 71

**Parasympathetic Activation:** 14, 21, 29, 32, 48, 55, 65, 68
