# Supplementary material for: Capturing women’s bodily experiences: Conception and formative stages of the women’s somatic experience inventory
Source: PLoS One. 2026 Jul 13;21(7):e0353167. doi: 10.1371/journal.pone.0353167 (PMC13362119; doi:10.1371/journal.pone.0353167)
Supplement: Attachment — Submitted filename: Response to Reviewers.docx [file pone.0353167.s004.docx]

Thank you for reviewing PONE-D-25-18681, originally entitled, “*Developing a scale to capture how women experience their bodies: The Women’s Somatic Experience Inventory”.* Attached please find the substantially revised manuscript, renamed *Capturing Women’s Bodily Experiences: Conception and Formative Stages of the Women’s Somatic Experience Inventory* to align with reviewer and editor comments.

**REVISIONS TO ADDRESS JOURNAL REQUIREMENTS:**

**1. File naming structure was corrected.**

**2. New funding statement was included in our cover letter and is also presented below.**

*This work was supported by the National Institutes of Health [National Institute on Alcohol Abuse Alcoholism; R01 AA027017 (JFB); F31 AA030178 (KLP);* [*https://www.niaaa.nih.gov/*](https://nam02.safelinks.protection.outlook.com/?url=https%3A%2F%2Fwww.niaaa.nih.gov%2F&data=05%7C02%7Cjbuckman%40kines.rutgers.edu%7C4c9039b05b12402430e708de0d75e056%7Cb92d2b234d35447093ff69aca6632ffe%7C1%7C0%7C638962997424958937%7CUnknown%7CTWFpbGZsb3d8eyJFbXB0eU1hcGkiOnRydWUsIlYiOiIwLjAuMDAwMCIsIlAiOiJXaW4zMiIsIkFOIjoiTWFpbCIsIldUIjoyfQ%3D%3D%7C0%7C%7C%7C&sdata=T7rBvQNzvn8vQmOjiBo%2F3yQFFjhIYth3vp2v6kGFoJA%3D&reserved=0)*] and a Society of Addiction Psychology (American Psychological Association Division 50;* [*https://addictionpsychology.org/*](https://nam02.safelinks.protection.outlook.com/?url=https%3A%2F%2Faddictionpsychology.org%2F&data=05%7C02%7Cjbuckman%40kines.rutgers.edu%7C4c9039b05b12402430e708de0d75e056%7Cb92d2b234d35447093ff69aca6632ffe%7C1%7C0%7C638962997424980327%7CUnknown%7CTWFpbGZsb3d8eyJFbXB0eU1hcGkiOnRydWUsIlYiOiIwLjAuMDAwMCIsIlAiOiJXaW4zMiIsIkFOIjoiTWFpbCIsIldUIjoyfQ%3D%3D%7C0%7C%7C%7C&sdata=dfqneYZucK8r726kCjFpqI5g0xaK%2FnGEJGpMK5iV7KQ%3D&reserved=0)*) Student Research Grant. There was no additional external funding received for this study. The funders had no role in study design, data collection and analysis, decision to publish, or preparation of the manuscript.*

**3. Including the phrase “data not shown” in the manuscript.**

The point related to this phrase as not relevant to the study and, in our process of simplifying the manuscript, as requested by the reviewers, we removed this statement.

**4. Captions for Supporting Information files** are now included.

**RESPONSE TO REVIEWS:**

**Title, abstract and introduction** **- strong emphasis on the menstrual cycle, rather than general focus on women’s bodily experience**

- We thank the reviewer for this comment and agree that the potential for this scale goes beyond menstrual health. We have revised the title and made revisions throughout the document to expand the focus to more global bodily experiences of women.

**Methods - excessively discursive**

- We appreciate this comment and, in retrospect, agreed that there was far too much detail. Please find a significantly streamlined method section.

**Methods - report the internal consistency of the instruments used and specifying how the scores obtained on each scale should be interpreted, clarifying what higher or lower scores mean.**

- We now include Cronbach’s alpha scores for the PSST and BAQ. We also have clarified in the Methods and Results how each scale score should be interpreted.

**Methods - choice of sample is a limitation and the lifestyles of emerging adults makes it difficult to fully support the associations observed between bodily experiences and behaviors.**

- We fully agree that emerging adulthood is a specific and time-limited life stage. The adoption and maintenance of healthy (exercise) and unhealthy (substance use) behaviors at this stage, and how these behaviors crystallize into habit to affect lifelong health and quality of life is a main focus of our lab. Our goal in including associations between somatic experiences and behaviors in this study was solely for proof-of-concept and as a first step in demonstrating convergent validity of the identified subscales. We note that the subscales identified in this manuscript represents the broad features and valence of somatic states; it does not present a finalized or validated scale; additional samples and the use of the refined item list we report are still needed. We have carefully reviewed the manuscript to ensure we do not overstate the results and add this point to the discussion limitation.

**Results - the reported fit indices fall below the thresholds commonly considered acceptable**.

- This is now added to the limitations. We acknowledge that these fit indices would be considered low for confirmatory analyses, but are in line with the conceptualization/preliminary creation goals of this paper wherein we report the process of creating a comprehensive list and pruning the list to a more tractable set of items (rather than creating a final survey). Our goal is to inspire other researchers to consider this starting point and help us push forward the concepts presented to promote the science of women.

**Results - no clear guidance on how to interpret the subscale scores**.

- We now clarify in Section 3.5 (and throughout the manuscript) that higher factor scores imply more frequent experiences in this domain. Thus, more frequent negative somatic experiences were associated with more premenstrual symptom severity. Interestingly, the analyses also suggest that the frequency of Physical Intimacy and Activated & Attractive somatic experiences was higher in hormonal contraception users. These analyses are meant only as proof-of-concept.

**Conclusions - expand discussion to address the potential clinical and applied relevance of the scale**

- Throughout the discussion, we have added statement that illustrate how the scale, once further developed and tested, could be used in research and in clinical settings.
