# Supplementary material for: Capturing women’s bodily experiences: Conception and formative stages of the women’s somatic experience inventory
Source: PLoS One. 2026 Jul 13;21(7):e0353167. doi: 10.1371/journal.pone.0353167 (PMC13362119; doi:10.1371/journal.pone.0353167)
Supplement: Attachment — Submitted filename: WSEI Revision 2 Response to Reviews FINAL.docx [file pone.0353167.s005.docx]

*Please submit your revised manuscript by Mar 28 2026 11:59PM.*

We appreciate the thoughtful comments from reviewers and believe the revisions have strengthened the manuscript. We have carefully reorganized certain sections, reframed concepts, and clarified topics to improve transparency and flow of the manuscript. We respond to each reviewer’s comments in detail below.

**Reviewer #1: 
…a more systematic distinction between questionnaire (when referring specifically to the ad hoc instrument developed in this study) and survey (when referring to the overall online booklet, which includes multiple blocks of questions in addition to the somatic experience questionnaire).**

We have carefully reframed the manuscript to improve flow and enhance transparency of what was done, when. We emphasize that this study is a first step towards the goal of developing a Women’s Somatic Experience Inventory; it has not yet been developed.

In this manuscript, we identify the most promising items and factors and provide initial validation for them. Throughout the manuscript, we now focus clearly on the survey results, referencing the inventory as a future goal. We have also used more precise language when describing the analyses and results, specifying “items”, “clusters”, and “factors” at each step; we also removed the use of “subscales”.

**…adding a dedicated subsection in which the study aims and any hypotheses are explicitly and clearly described. This would help the reader better understand the rationale guiding the subsequent analyses.**

We have added detailed hypotheses at the end of the introduction, which we believe helped the flow of the paper, clearly delineating the goals as: finding the items, creating factors, and providing preliminary validation for the factors. We separate a priori hypotheses about identifying factors and post hoc hypotheses that arose after the factors were identified.

**…reorganizing the Participants and Procedure sections. The manuscript reports that 310 participants were recruited, while the data presented refer to 300 participants. One possible solution would be to merge these sections into a single paragraph titled Procedure and Participants, clearly describing the recruitment and inclusion flow.**

The reasons for excluding 10 participants from analysis has been added in the Participants section. See p. 6
 **…In the description of the Premenstrual Symptoms Screening Tool, the instrument is initially described as consisting of two sections, but only the section used in the present study is subsequently described. I suggest explicitly clarifying this point to avoid unmet expectations for the reader.**

We had provided this information in the methods but, during this revision and in response to other critiques, we added a description of the PSST to the introduction. This allowed us to more thoroughly describe the BAQ and PSST, why they were chosen, and how they were used. Included in the new PSST paragraph is the explicit mention of using only the first section of the screener. See p. 4

**In the section describing the somatic items, additional details regarding the refinement process would be helpful. In particular, it would be important to clarify who the five individuals external to the research team were, their background, how they were selected, and the criteria or procedures they used to provide feedback on item refinement.**

This has been clarified. This was a very early step that was meant more as ‘proofing’ than conceptually influencing the survey. The study PI and lab Director made all final decisions. See p. 12
 **The distinction between the Data Analysis and Results sections is currently unclear. The Data Analysis section should describe only the statistical procedures used (including relevant indices and cut-off values for interpretation), whereas some results appear to be anticipated and to guide subsequent analyses. I suggest relocating these contents to the Results section.**

We had a difficult time determining where the Method ended and the Results began, which is not uncommon in methodologically focused papers. In this revision, we restructured the paper to flow better, following the hypotheses in the introduction. We believe that this separation of information is more logical.

**…organizing the Results section into subsections that explicitly reflect each statistical analysis conducted in relation to the specific study aims, which would substantially improve overall readability.**

As noted above, we restructured the paper and improved our subheading names to allow analyses and results to flow more easily from the hypotheses. The aims are (a) identify and refine a comprehensive list, (b) identify and validate factors, (c) provide proof of concept.

**…identifying the key take-home messages of the work and reorganizing the Discussion around the primary results, as the current structure risks being redundant.**

We have restructured and shortened the discussion to parallel the new flow of the hypotheses and results.

**In several passages, the observed associations are interpreted as supporting underlying constructs or mechanisms, despite the study being explicitly positioned as formative and exploratory in the development of the instrument. It would be advisable to align the level of interpretation more closely with the preliminary nature of the work by adopting more cautious language.**

We have carefully assessed the revised discussion to ensure insufficiently cautious language has been removed.

**Some theoretical interpretations appear to be constructed post hoc and are not always clearly linked to previously stated aims or hypotheses. Clarifying the study objectives more explicitly and subsequently calibrating the interpretations accordingly would strengthen the coherence of the manuscript.**

We now present clear aims and hypotheses in the introduction. The methods and results were reorganized. The discussion was shortened and interpretations are more tightly conceptually linked to the aims.

**Although a limitations section is included, it would be helpful to clarify more explicitly how these limitations should guide the interpretation of the results.**

This has been added to the limitation section.

**Finally, the applied and clinical implications are discussed rather broadly given the current stage of development and validation of the instrument. It may be useful to scale back these conclusions or to more clearly reiterate the preliminary nature of the findings.**

We agree; these were added in a prior round of revisions but move beyond the preliminary nature of this study.

**Reviewer #2: 
Introduction
• Although the somatic marker hypothesis is explained very well in the introduction section, for the validated questionnaires and measures that the writers used to investigate associations with WSEI, specific literature providing results of previous studies could be mentioned in the introduction. Briefly summarizing key findings from previous studies would help clarify why these measures were selected and how they conceptually relate to the WSEI.**

We have shifted the description of the BAQ and PSST to the introduction and added more details from the literature to better explain why these measures were selected.

**Results
The writers may consider moving some of the main-text tables into the supplementary materials to improve clarity.**

We now have 5 tables and 3 figures in the body of the manuscript. Tables have been merged, reformatted and often moved to supplementary materials. The two new figures highlight the main findings from 4 tables, which are now condensed to 2 tables in supplemental materials.

**Discussion
Proof-of-concept analyses: The subsections of this section may be improved by providing information on how current results relate to past studies that were conducted using these tools.**

We have added details to the introduction about past studies with BAQ and PSST and added specific hypotheses. Now, in the discussion, we also point to prior findings related to body awareness, menstrual symptoms, and exercise in relation to our results.

**The descriptions of the questionnaires such as: “The items that comprise the Body Awareness Questionnaire (BAQ) gauge attentiveness to general physiological states and expectations from those states (e.g., “I can tell when I go to bed how well I will sleep that night.”). Items cover awareness of states like fatigue and illness as well as body-specific responsivity (e.g., GI) concerning health behaviors like sleep, exercise, and eating” might be better suited for the methods section.**

We have reorganized all information related to BAQ, including in the introduction, method and discussion.
 **The use of the term “qualitative techniques" (also present in the introduction) may be misleading as the study does not involve qualitative data. Clarifying this may improve methodological transparency.**

We have removed the term in favor of more accurate descriptions, such as “concept- and theory-driven techniques”.
