# Supplementary material for: Capturing women’s bodily experiences: Conception and formative stages of the women’s somatic experience inventory
Source: PLoS One. 2026 Jul 13;21(7):e0353167. doi: 10.1371/journal.pone.0353167 (PMC13362119; doi:10.1371/journal.pone.0353167)
Supplement: Peer review history

# Peer review: Decision Letter 0

**DOI:** 10.1371/journal.pone.0353167.r001

**Authors:** Stefano Federici

17 Oct 2025

PONE-D-25-18681

Developing a scale to capture how women experience their bodies: The Women’s Somatic Experience Inventory

PLOS ONE

Dear Dr. Piersol,

Thank you for submitting your manuscript to PLOS ONE. After careful consideration, we feel that it has merit but does not fully meet PLOS ONE’s publication criteria as it currently stands. Therefore, we invite you to submit a revised version of the manuscript that addresses the points raised during the review process.

==============================

- The reviewer appreciates the originality and methodological rigor of the study but identifies several areas for improvement. Specifically, the abstract and introduction overemphasize the menstrual cycle, potentially narrowing the perceived scope of the instrument. The reviewer suggests clarifying that the questionnaire captures general bodily experiences in women, not limited to the menstrual cycle, and recommends a clearer, more representative title.
- The methods section is praised for its detail but could be made more readable by restructuring the text, reporting internal consistency indices (e.g., Cronbach’s α), and clarifying score interpretation. The sample, limited to young university women, restricts generalizability and should be discussed as a limitation.
- Fit indices (CFI, TLI) are below conventional cut-offs, and this should be acknowledged and discussed. The reviewer also calls for clearer interpretation of subscales and for an expanded discussion on clinical and applied implications.

==============================

Please submit your revised manuscript by Dec 01 2025 11:59PM. If you will need more time than this to complete your revisions, please reply to this message or contact the journal office at plosone@plos.org. When you're ready to submit your revision, log on to https://www.editorialmanager.com/pone/ and select the 'Submissions Needing Revision' folder to locate your manuscript file.

Please include the following items when submitting your revised manuscript:

- A rebuttal letter that responds to each point raised by the academic editor and reviewer(s). You should upload this letter as a separate file labeled 'Response to Reviewers'.
- A marked-up copy of your manuscript that highlights changes made to the original version. You should upload this as a separate file labeled 'Revised Manuscript with Track Changes'.
- An unmarked version of your revised paper without tracked changes. You should upload this as a separate file labeled 'Manuscript'.

If you would like to make changes to your financial disclosure, please include your updated statement in your cover letter. Guidelines for resubmitting your figure files are available below the reviewer comments at the end of this letter.

If applicable, we recommend that you deposit your laboratory protocols in protocols.io to enhance the reproducibility of your results. Protocols.io assigns your protocol its own identifier (DOI) so that it can be cited independently in the future. For instructions see: https://journals.plos.org/plosone/s/submission-guidelines#loc-laboratory-protocols. Additionally, PLOS ONE offers an option for publishing peer-reviewed Lab Protocol articles, which describe protocols hosted on protocols.io. Read more information on sharing protocols at https://plos.org/protocols?utm_medium=editorial-email&utm_source=authorletters&utm_campaign=protocols.

We look forward to receiving your revised manuscript.

Kind regards,

Stefano Federici, Ph.D.

Academic Editor

PLOS ONE

**Journal Requirements:**

1. When submitting your revision, we need you to address these additional requirements.

Please ensure that your manuscript meets PLOS ONE's style requirements, including those for file naming. The PLOS ONE style templates can be found at

https://journals.plos.org/plosone/s/file?id=wjVg/PLOSOne_formatting_sample_main_body.pdf and

https://journals.plos.org/plosone/s/file?id=ba62/PLOSOne_formatting_sample_title_authors_affiliations.pdf

2. Thank you for stating in your Funding Statement:

This work was supported by the National Institutes of Health [National Institute on Alcohol Abuse Alcoholism; R01 AA027017 (JFB); F31 AA030178 (KLP); https://www.niaaa.nih.gov/] and a Society of Addiction Psychology (American Psychological Association Division 50; https://addictionpsychology.org/) Student Research Grant. The funders had no role in study design, data collection and analysis, decision to publish, or preparation of the manuscript.

Please provide an amended statement that declares *all* the funding or sources of support (whether external or internal to your organization) received during this study, as detailed online in our guide for authors at http://journals.plos.org/plosone/s/submit-now.  Please also include the statement “There was no additional external funding received for this study.” in your updated Funding Statement.

Please include your amended Funding Statement within your cover letter. We will change the online submission form on your behalf.

3. We note that you have included the phrase “data not shown” in your manuscript. Unfortunately, this does not meet our data sharing requirements. PLOS does not permit references to inaccessible data. We require that authors provide all relevant data within the paper, Supporting Information files, or in an acceptable, public repository. Please add a citation to support this phrase or upload the data that corresponds with these findings to a stable repository (such as Figshare or Dryad) and provide and URLs, DOIs, or accession numbers that may be used to access these data. Or, if the data are not a core part of the research being presented in your study, we ask that you remove the phrase that refers to these data.

4. Please include captions for your Supporting Information files at the end of your manuscript, and update any in-text citations to match accordingly. Please see our Supporting Information guidelines for more information: http://journals.plos.org/plosone/s/supporting-information.

5. If the reviewer comments include a recommendation to cite specific previously published works, please review and evaluate these publications to determine whether they are relevant and should be cited. There is no requirement to cite these works unless the editor has indicated otherwise.

**Additional Editor Comments:**

The reviewer appreciates the originality and methodological rigor of the study but identifies several areas for improvement. Specifically, the abstract and introduction overemphasize the menstrual cycle, potentially narrowing the perceived scope of the instrument. The reviewer suggests clarifying that the questionnaire captures general bodily experiences in women, not limited to the menstrual cycle, and recommends a clearer, more representative title.

The methods section is praised for its detail but could be made more readable by restructuring the text, reporting internal consistency indices (e.g., Cronbach’s α), and clarifying score interpretation. The sample, limited to young university women, restricts generalizability and should be discussed as a limitation.

Fit indices (CFI, TLI) are below conventional cut-offs, and this should be acknowledged and discussed. The reviewer also calls for clearer interpretation of subscales and for an expanded discussion on clinical and applied implications.

[Note: HTML markup is below. Please do not edit.]

Reviewers' comments:

Reviewer's Responses to Questions

**Comments to the Author**

1. Is the manuscript technically sound, and do the data support the conclusions?

The manuscript must describe a technically sound piece of scientific research with data that supports the conclusions. Experiments must have been conducted rigorously, with appropriate controls, replication, and sample sizes. The conclusions must be drawn appropriately based on the data presented.

Reviewer #1: Yes

**********

2. Has the statistical analysis been performed appropriately and rigorously?

Reviewer #1: Yes

**********

3. Have the authors made all data underlying the findings in their manuscript fully available?

The PLOS Data policy requires authors to make all data underlying the findings described in their manuscript fully available without restriction, with rare exception (please refer to the Data Availability Statement in the manuscript PDF file). The data should be provided as part of the manuscript or its supporting information, or deposited to a public repository. For example, in addition to summary statistics, the data points behind means, medians and variance measures should be available. If there are restrictions on publicly sharing data—e.g. participant privacy or use of data from a third party—those must be specified.

Reviewer #1: Yes

**********

4. Is the manuscript presented in an intelligible fashion and written in standard English?

PLOS ONE does not copyedit accepted manuscripts, so the language in submitted articles must be clear, correct, and unambiguous. Any typographical or grammatical errors should be corrected at revision, so please note any specific errors here.

Reviewer #1: Yes

**********

5. Review Comments to the Author

Please use the space provided to explain your answers to the questions above. You may also include additional comments for the author, including concerns about dual publication, research ethics, or publication ethics. (Please upload your review as an attachment if it exceeds 20,000 characters)

**Reviewer #1:** I thank the editor for giving me the opportunity to review this manuscript, which represents an original and methodologically rigorous contribution. However, there are several aspects that deserve attention.

With regard to the abstract and introduction, I noticed a strong emphasis on the menstrual cycle, which may give the impression that the instrument was developed exclusively to assess bodily experiences related to that period. In reality, the questionnaire measures a broader range of somatic experiences, not limited to the menstrual cycle. I would therefore suggest reformulating these sections to clarify from the outset that the questionnaire was conceived as a general measure of women’s bodily experience, with the menstrual cycle as a possible area of application but not the only or the primary one. In this perspective, I would also recommend revising the title, opting for a more direct formulation consistent with the aims of the study, such as Capturing Women’s Bodily Experience: Development and Validation of the Women’s Somatic Experience Inventory, which makes explicit that the study involves both the development and the validation of the new scale.

The methods section is very detailed, which is a strength as it favors the replicability of the study. However, the section on measures is in some places excessively discursive, with very long paragraphs that could be restructured to improve readability. A simple adjustment could be to highlight in bold the names of the questionnaires used. Furthermore, I would recommend reporting the internal consistency of the instruments in the sample (e.g., Cronbach’s α values) and specifying how the scores obtained on each scale should be interpreted, clarifying what higher or lower scores mean.

The choice of sample is another limitation to consider: the exclusive focus on young university women aged 18 to 25 reduces the generalizability of the results. This aspect is particularly relevant because emerging adulthood is a life stage characterized by significant developmental changes and the adoption of risk behaviors, such as alcohol consumption or irregular lifestyles, which can strongly influence both well-being and bodily perception. Without controlling for variables specific to this life stage, it is difficult to fully support the associations observed between bodily experiences and behaviors; it would be advisable to include such variables in the analyses or to discuss this limitation explicitly.

In the results section, the reported fit indices for the factorial models (CFI and TLI) fall below the thresholds commonly considered acceptable in the literature (≥ .95). It is important to highlight this limitation and discuss its implications for the robustness of the scale. In addition, there is currently no clear guidance on how to interpret the subscale scores: there do not appear to be indications of what higher or lower values mean, which reduces the immediacy and practical utility of the instrument.

Finally, in the conclusions I would suggest expanding the discussion to address the potential clinical and applied relevance of the scale. At present, the emphasis is placed almost exclusively on the methodological value of the instrument, but discussing how it could be used in clinical practice, in women’s health research, or in the prevention of risk behaviors would strengthen both its impact and its interest.

**********

6. PLOS authors have the option to publish the peer review history of their article (what does this mean?). If published, this will include your full peer review and any attached files.

If you choose “no”, your identity will remain anonymous but your review may still be made public.

**Do you want your identity to be public for this peer review?**  For information about this choice, including consent withdrawal, please see our Privacy Policy.

Reviewer #1: No

**********

[NOTE: If reviewer comments were submitted as an attachment file, they will be attached to this email and accessible via the submission site. Please log into your account, locate the manuscript record, and check for the action link "View Attachments". If this link does not appear, there are no attachment files.]

While revising your submission, please upload your figure files to the Preflight Analysis and Conversion Engine (PACE) digital diagnostic tool, https://pacev2.apexcovantage.com/. PACE helps ensure that figures meet PLOS requirements. To use PACE, you must first register as a user. Registration is free. Then, login and navigate to the UPLOAD tab, where you will find detailed instructions on how to use the tool. If you encounter any issues or have any questions when using PACE, please email PLOS at figures@plos.org. Please note that Supporting Information files do not need this step.

# Author comment: Author response to Decision Letter 1

**DOI:** 10.1371/journal.pone.0353167.r002

15 Dec 2025

Thank you for reviewing PONE-D-25-18681, originally entitled, “Developing a scale to capture how women experience their bodies: The Women’s Somatic Experience Inventory”. Attached please find the substantially revised manuscript, renamed Capturing Women’s Bodily Experiences: Conception and Formative Stages of the Women’s Somatic Experience Inventory to align with reviewer and editor comments.

REVISIONS TO ADDRESS JOURNAL REQUIREMENTS:

1. File naming structure was corrected.

2. New funding statement was included in our cover letter and is also presented below.

This work was supported by the National Institutes of Health [National Institute on Alcohol Abuse Alcoholism; R01 AA027017 (JFB); F31 AA030178 (KLP); https://www.niaaa.nih.gov/] and a Society of Addiction Psychology (American Psychological Association Division 50; https://addictionpsychology.org/) Student Research Grant. There was no additional external funding received for this study. The funders had no role in study design, data collection and analysis, decision to publish, or preparation of the manuscript.

3. Including the phrase “data not shown” in the manuscript.

The point related to this phrase as not relevant to the study and, in our process of simplifying the manuscript, as requested by the reviewers, we removed this statement.

4. Captions for Supporting Information files are now included.

RESPONSE TO REVIEWS:

Title, abstract and introduction - strong emphasis on the menstrual cycle, rather than general focus on women’s bodily experience

• We thank the reviewer for this comment and agree that the potential for this scale goes beyond menstrual health. We have revised the title and made revisions throughout the document to expand the focus to more global bodily experiences of women.

Methods - excessively discursive

• We appreciate this comment and, in retrospect, agreed that there was far too much detail. Please find a significantly streamlined method section.

Methods - report the internal consistency of the instruments used and specifying how the scores obtained on each scale should be interpreted, clarifying what higher or lower scores mean.

• We now include Cronbach’s alpha scores for the PSST and BAQ. We also have clarified in the Methods and Results how each scale score should be interpreted.

Methods - choice of sample is a limitation and the lifestyles of emerging adults makes it difficult to fully support the associations observed between bodily experiences and behaviors.

• We fully agree that emerging adulthood is a specific and time-limited life stage. The adoption and maintenance of healthy (exercise) and unhealthy (substance use) behaviors at this stage, and how these behaviors crystallize into habit to affect lifelong health and quality of life is a main focus of our lab. Our goal in including associations between somatic experiences and behaviors in this study was solely for proof-of-concept and as a first step in demonstrating convergent validity of the identified subscales. We note that the subscales identified in this manuscript represents the broad features and valence of somatic states; it does not present a finalized or validated scale; additional samples and the use of the refined item list we report are still needed. We have carefully reviewed the manuscript to ensure we do not overstate the results and add this point to the discussion limitation.

Results - the reported fit indices fall below the thresholds commonly considered acceptable.

• This is now added to the limitations. We acknowledge that these fit indices would be considered low for confirmatory analyses, but are in line with the conceptualization/preliminary creation goals of this paper wherein we report the process of creating a comprehensive list and pruning the list to a more tractable set of items (rather than creating a final survey). Our goal is to inspire other researchers to consider this starting point and help us push forward the concepts presented to promote the science of women.

Results - no clear guidance on how to interpret the subscale scores.

• We now clarify in Section 3.5 (and throughout the manuscript) that higher factor scores imply more frequent experiences in this domain. Thus, more frequent negative somatic experiences were associated with more premenstrual symptom severity. Interestingly, the analyses also suggest that the frequency of Physical Intimacy and Activated & Attractive somatic experiences was higher in hormonal contraception users. These analyses are meant only as proof-of-concept.

Conclusions - expand discussion to address the potential clinical and applied relevance of the scale

• Throughout the discussion, we have added statement that illustrate how the scale, once further developed and tested, could be used in research and in clinical settings.

Attachment Submitted filename: Response to Reviewers.docx

# Peer review: Decision Letter 1

**DOI:** 10.1371/journal.pone.0353167.r003

**Authors:** Stefano Federici, Domna Banakou

11 Feb 2026

PONE-D-25-18681R1

Capturing women's bodily experiences: Conception and formative stages of the Women's Somatic Experience Inventory

PLOS One

Dear Dr. Piersol,

Thank you for submitting your manuscript to PLOS ONE. After careful consideration, we feel that it has merit but does not fully meet PLOS ONE’s publication criteria as it currently stands. Therefore, we invite you to submit a revised version of the manuscript that addresses the points raised during the review process.

The reviewers acknowledge the manuscript as well written and promising, but request revisions to improve clarity, structure, and methodological transparency. For example, it is recommended to clearly distinguish “questionnaire” vs. “survey,” add an explicit aims/hypotheses subsection, reconcile participant numbers, and reorganize Participants and Procedure, clarifying tools descriptions, and providing more detail on the somatic item refinement process (including who provided external feedback and how). The Data Analysis should be clearly separated from the Results, reorganizing Results into aim-linked subsections, and revising the Discussion to reduce repetition, emphasize key contributions, relate findings to prior literature, use more cautious language consistent with an exploratory/early validation study, and scale back broad clinical implications while sharpening how limitations constrain interpretation. Kindly refer to the detailed comments below for the revised manuscript.

Please submit your revised manuscript by Mar 28 2026 11:59PM. If you will need more time than this to complete your revisions, please reply to this message or contact the journal office at plosone@plos.org. When you're ready to submit your revision, log on to https://www.editorialmanager.com/pone/ and select the 'Submissions Needing Revision' folder to locate your manuscript file.

Please include the following items when submitting your revised manuscript:

- A letter that responds to each point raised by the academic editor and reviewer(s). You should upload this letter as a separate file labeled 'Response to Reviewers'.
- A marked-up copy of your manuscript that highlights changes made to the original version. You should upload this as a separate file labeled 'Revised Manuscript with Track Changes'.
- An unmarked version of your revised paper without tracked changes. You should upload this as a separate file labeled 'Manuscript'.

If you would like to make changes to your financial disclosure, please include your updated statement in your cover letter. Guidelines for resubmitting your figure files are available below the reviewer comments at the end of this letter.

If applicable, we recommend that you deposit your laboratory protocols in protocols.io to enhance the reproducibility of your results. Protocols.io assigns your protocol its own identifier (DOI) so that it can be cited independently in the future. For instructions see: https://journals.plos.org/plosone/s/submission-guidelines#loc-laboratory-protocols. Additionally, PLOS ONE offers an option for publishing peer-reviewed Lab Protocol articles, which describe protocols hosted on protocols.io. Read more information on sharing protocols at https://plos.org/protocols?utm_medium=editorial-email&utm_source=authorletters&utm_campaign=protocols.

We look forward to receiving your revised manuscript.

Kind regards,

Domna Banakou

Academic Editor

PLOS One

Journal Requirements:

If the reviewer comments include a recommendation to cite specific previously published works, please review and evaluate these publications to determine whether they are relevant and should be cited. There is no requirement to cite these works unless the editor has indicated otherwise.

Reviewers' comments:

Reviewer's Responses to Questions

**Comments to the Author**

1. If the authors have adequately addressed your comments raised in a previous round of review and you feel that this manuscript is now acceptable for publication, you may indicate that here to bypass the “Comments to the Author” section, enter your conflict of interest statement in the “Confidential to Editor” section, and submit your "Accept" recommendation.

Reviewer #1: (No Response)

Reviewer #2: (No Response)

**********

2. Is the manuscript technically sound, and do the data support the conclusions?

The manuscript must describe a technically sound piece of scientific research with data that supports the conclusions. Experiments must have been conducted rigorously, with appropriate controls, replication, and sample sizes. The conclusions must be drawn appropriately based on the data presented.

Reviewer #1: Partly

Reviewer #2: Yes

**********

3. Has the statistical analysis been performed appropriately and rigorously?

Reviewer #1: Yes

Reviewer #2: Yes

**********

4. Have the authors made all data underlying the findings in their manuscript fully available?

The PLOS Data policy requires authors to make all data underlying the findings described in their manuscript fully available without restriction, with rare exception (please refer to the Data Availability Statement in the manuscript PDF file). The data should be provided as part of the manuscript or its supporting information, or deposited to a public repository. For example, in addition to summary statistics, the data points behind means, medians and variance measures should be available. If there are restrictions on publicly sharing data—e.g. participant privacy or use of data from a third party—those must be specified.

Reviewer #1: Yes

Reviewer #2: Yes

**********

5. Is the manuscript presented in an intelligible fashion and written in standard English?

PLOS ONE does not copyedit accepted manuscripts, so the language in submitted articles must be clear, correct, and unambiguous. Any typographical or grammatical errors should be corrected at revision, so please note any specific errors here.

Reviewer #1: Yes

Reviewer #2: Yes

**********

6. Review Comments to the Author

Please use the space provided to explain your answers to the questions above. You may also include additional comments for the author, including concerns about dual publication, research ethics, or publication ethics. (Please upload your review as an attachment if it exceeds 20,000 characters)

Reviewer #1: I thank the authors for revising the manuscript in line with the suggestions provided during the first round of review. After carefully re-reading the revised version, I believe that several issues still require clarification in order to improve the overall clarity, methodological transparency, and readiness of the manuscript for publication. Below I outline a number of points that I consider important to address.

In the manuscript, I would recommend a more systematic distinction between questionnaire (when referring specifically to the ad hoc instrument developed in this study) and survey (when referring to the overall online booklet, which includes multiple blocks of questions in addition to the somatic experience questionnaire).

I suggest adding a dedicated subsection in which the study aims and any hypotheses are explicitly and clearly described. This would help the reader better understand the rationale guiding the subsequent analyses.

I would recommend reorganizing the Participants and Procedure sections. The manuscript reports that 310 participants were recruited, while the data presented refer to 300 participants. One possible solution would be to merge these sections into a single paragraph titled Procedure and Participants, clearly describing the recruitment and inclusion flow.

In the description of the Premenstrual Symptoms Screening Tool, the instrument is initially described as consisting of two sections, but only the section used in the present study is subsequently described. I suggest explicitly clarifying this point to avoid unmet expectations for the reader.

In the section describing the somatic items, additional details regarding the refinement process would be helpful. In particular, it would be important to clarify who the five individuals external to the research team were, their background, how they were selected, and the criteria or procedures they used to provide feedback on item refinement.

The distinction between the Data Analysis and Results sections is currently unclear. The Data Analysis section should describe only the statistical procedures used (including relevant indices and cut-off values for interpretation), whereas some results appear to be anticipated and to guide subsequent analyses. I suggest relocating these contents to the Results section.

Finally, I recommend organizing the Results section into subsections that explicitly reflect each statistical analysis conducted in relation to the specific study aims, which would substantially improve overall readability.

The Discussion section is partly descriptive and tends to reiterate the results, without clearly distinguishing the main contributions of the study from more secondary or exploratory findings. I suggest explicitly identifying the key take-home messages of the work and reorganizing the Discussion around the primary results, as the current structure risks being redundant.

In several passages, the observed associations are interpreted as supporting underlying constructs or mechanisms, despite the study being explicitly positioned as formative and exploratory in the development of the instrument. It would be advisable to align the level of interpretation more closely with the preliminary nature of the work by adopting more cautious language.

Some theoretical interpretations appear to be constructed post hoc and are not always clearly linked to previously stated aims or hypotheses. Clarifying the study objectives more explicitly and subsequently calibrating the interpretations accordingly would strengthen the coherence of the manuscript.

Although a limitations section is included, it would be helpful to clarify more explicitly how these limitations should guide the interpretation of the results.

Finally, the applied and clinical implications are discussed rather broadly given the current stage of development and validation of the instrument. It may be useful to scale back these conclusions or to more clearly reiterate the preliminary nature of the findings.

Reviewer #2: Capturing Women’s Bodily Experiences: Women’s Somatic Experience Inventory is a clearly written and thoughtfully designed manuscript that presents the development of a novel instrument to assess female-specific somatic experiences and their associations with bodily awareness, health-related behaviors, premenstrual symptom severity and hormonal contraception use. The proposed inventory has the potential to be a valuable tool for future clinical and research applications.

Recommendations:

Introduction

• Although the somatic marker hypothesis is explained very well in the introduction section, for the validated questionnaires and measures that the writers used to investigate associations with WSEI, specific literature providing results of previous studies could be mentioned in the introduction. Briefly summarizing key findings from previous studies would help clarify why these measures were selected and how they conceptually relate to the WSEI.

Results

• The writers may consider moving some of the main-text tables into the supplementary materials to improve clarity.

Discussion

• Proof-of-concept analyses: The subsections of this section may be improved by providing information on how current results relate to past studies that were conducted using these tools.

• The descriptions of the questionnaires such as: “The items that comprise the Body Awareness Questionnaire (BAQ) gauge attentiveness to general physiological states and expectations from those states (e.g., “I can tell when I go to bed how well I will sleep that night.”). Items cover awareness of states like fatigue and illness as well as body-specific responsivity (e.g., GI) concerning health behaviors like sleep, exercise, and eating” might be better suited for the methods section.

• The use of the term “qualitative techniques" (also present in the introduction) may be misleading as the study does not involve qualitative data. Clarifying this may improve methodological transparency.

**********

7. PLOS authors have the option to publish the peer review history of their article (what does this mean?). If published, this will include your full peer review and any attached files.

If you choose “no”, your identity will remain anonymous but your review may still be made public.

**Do you want your identity to be public for this peer review?**  For information about this choice, including consent withdrawal, please see our Privacy Policy.

Reviewer #1: **Yes:** Livia Buratta

Reviewer #2: No

**********

[NOTE: If reviewer comments were submitted as an attachment file, they will be attached to this email and accessible via the submission site. Please log into your account, locate the manuscript record, and check for the action link "View Attachments". If this link does not appear, there are no attachment files.]

To ensure your figures meet our technical requirements, please review our figure guidelines: https://journals.plos.org/plosone/s/figures

You may also use PLOS’s free figure tool, NAAS, to help you prepare publication quality figures: https://journals.plos.org/plosone/s/figures#loc-tools-for-figure-preparation.

NAAS will assess whether your figures meet our technical requirements by comparing each figure against our figure specifications.

# Author comment: Author response to Decision Letter 2

**DOI:** 10.1371/journal.pone.0353167.r004

28 Mar 2026

We appreciate the thoughtful comments from reviewers and believe the revisions have strengthened the manuscript. We have carefully reorganized certain sections, reframed concepts, and clarified topics to improve transparency and flow of the manuscript. We respond to each reviewer’s comments in detail below.

Reviewer #1:

…a more systematic distinction between questionnaire (when referring specifically to the ad hoc instrument developed in this study) and survey (when referring to the overall online booklet, which includes multiple blocks of questions in addition to the somatic experience questionnaire).

We have carefully reframed the manuscript to improve flow and enhance transparency of what was done, when. We emphasize that this study is a first step towards the goal of developing a Women’s Somatic Experience Inventory; it has not yet been developed.

In this manuscript, we identify the most promising items and factors and provide initial validation for them. Throughout the manuscript, we now focus clearly on the survey results, referencing the inventory as a future goal. We have also used more precise language when describing the analyses and results, specifying “items”, “clusters”, and “factors” at each step; we also removed the use of “subscales”.

…adding a dedicated subsection in which the study aims and any hypotheses are explicitly and clearly described. This would help the reader better understand the rationale guiding the subsequent analyses.

We have added detailed hypotheses at the end of the introduction, which we believe helped the flow of the paper, clearly delineating the goals as: finding the items, creating factors, and providing preliminary validation for the factors. We separate a priori hypotheses about identifying factors and post hoc hypotheses that arose after the factors were identified.

…reorganizing the Participants and Procedure sections. The manuscript reports that 310 participants were recruited, while the data presented refer to 300 participants. One possible solution would be to merge these sections into a single paragraph titled Procedure and Participants, clearly describing the recruitment and inclusion flow.

The reasons for excluding 10 participants from analysis has been added in the Participants section. See p. 6

…In the description of the Premenstrual Symptoms Screening Tool, the instrument is initially described as consisting of two sections, but only the section used in the present study is subsequently described. I suggest explicitly clarifying this point to avoid unmet expectations for the reader.

We had provided this information in the methods but, during this revision and in response to other critiques, we added a description of the PSST to the introduction. This allowed us to more thoroughly describe the BAQ and PSST, why they were chosen, and how they were used. Included in the new PSST paragraph is the explicit mention of using only the first section of the screener. See p. 4

In the section describing the somatic items, additional details regarding the refinement process would be helpful. In particular, it would be important to clarify who the five individuals external to the research team were, their background, how they were selected, and the criteria or procedures they used to provide feedback on item refinement.

This has been clarified. This was a very early step that was meant more as ‘proofing’ than conceptually influencing the survey. The study PI and lab Director made all final decisions. See p. 12

The distinction between the Data Analysis and Results sections is currently unclear. The Data Analysis section should describe only the statistical procedures used (including relevant indices and cut-off values for interpretation), whereas some results appear to be anticipated and to guide subsequent analyses. I suggest relocating these contents to the Results section.

We had a difficult time determining where the Method ended and the Results began, which is not uncommon in methodologically focused papers. In this revision, we restructured the paper to flow better, following the hypotheses in the introduction. We believe that this separation of information is more logical.

…organizing the Results section into subsections that explicitly reflect each statistical analysis conducted in relation to the specific study aims, which would substantially improve overall readability.

As noted above, we restructured the paper and improved our subheading names to allow analyses and results to flow more easily from the hypotheses. The aims are (a) identify and refine a comprehensive list, (b) identify and validate factors, (c) provide proof of concept.

…identifying the key take-home messages of the work and reorganizing the Discussion around the primary results, as the current structure risks being redundant.

We have restructured and shortened the discussion to parallel the new flow of the hypotheses and results.

In several passages, the observed associations are interpreted as supporting underlying constructs or mechanisms, despite the study being explicitly positioned as formative and exploratory in the development of the instrument. It would be advisable to align the level of interpretation more closely with the preliminary nature of the work by adopting more cautious language.

We have carefully assessed the revised discussion to ensure insufficiently cautious language has been removed.

Some theoretical interpretations appear to be constructed post hoc and are not always clearly linked to previously stated aims or hypotheses. Clarifying the study objectives more explicitly and subsequently calibrating the interpretations accordingly would strengthen the coherence of the manuscript.

We now present clear aims and hypotheses in the introduction. The methods and results were reorganized. The discussion was shortened and interpretations are more tightly conceptually linked to the aims.

Although a limitations section is included, it would be helpful to clarify more explicitly how these limitations should guide the interpretation of the results.

This has been added to the limitation section.

Finally, the applied and clinical implications are discussed rather broadly given the current stage of development and validation of the instrument. It may be useful to scale back these conclusions or to more clearly reiterate the preliminary nature of the findings.

We agree; these were added in a prior round of revisions but move beyond the preliminary nature of this study.

Reviewer #2:

Introduction

• Although the somatic marker hypothesis is explained very well in the introduction section, for the validated questionnaires and measures that the writers used to investigate associations with WSEI, specific literature providing results of previous studies could be mentioned in the introduction. Briefly summarizing key findings from previous studies would help clarify why these measures were selected and how they conceptually relate to the WSEI.

We have shifted the description of the BAQ and PSST to the introduction and added more details from the literature to better explain why these measures were selected.

Results

The writers may consider moving some of the main-text tables into the supplementary materials to improve clarity.

We now have 5 tables and 3 figures in the body of the manuscript. Tables have been merged, reformatted and often moved to supplementary materials. The two new figures highlight the main findings from 4 tables, which are now condensed to 2 tables in supplemental materials.

Discussion

Proof-of-concept analyses: The subsections of this section may be improved by providing information on how current results relate to past studies that were conducted using these tools.

We have added details to the introduction about past studies with BAQ and PSST and added specific hypotheses. Now, in the discussion, we also point to prior findings related to body awareness, menstrual symptoms, and exercise in relation to our results.

The descriptions of the questionnaires such as: “The items that comprise the Body Awareness Questionnaire (BAQ) gauge attentiveness to general physiological states and expectations from those states (e.g., “I can tell when I go to bed how well I will sleep that night.”). Items cover awareness of states like fatigue and illness as well as body-specific responsivity (e.g., GI) concerning health behaviors like sleep, exercise, and eating” might be better suited for the methods section.

We have reorganized all information related to BAQ, including in the introduction, method and discussion.

The use of the term “qualitative techniques" (also present in the introduction) may be misleading as the study does not involve qualitative data. Clarifying this may improve methodological transparency.

We have removed the term in favor of more accurate descriptions, such as “concept- and theory-driven techniques”.

Attachment Submitted filename: WSEI Revision 2 Response to Reviews FINAL.docx

# Peer review: Decision Letter 2

**DOI:** 10.1371/journal.pone.0353167.r005

**Authors:** Stefano Federici, Domna Banakou, Domna Banakou

18 May 2026

PONE-D-25-18681R2

Capturing women's bodily experiences: Conception and formative stages of the Women's Somatic Experience Inventory

PLOS One

Dear Dr. Piersol,

Thank you for submitting your manuscript to PLOS ONE. After careful consideration, we feel that it has merit but does not fully meet PLOS ONE’s publication criteria as it currently stands. Therefore, we invite you to submit a revised version of the manuscript that addresses the points raised during the review process.

The manuscript is considerably improved and is now clearer about being an **early-stage, formative inventory-development study** , rather than a completed and fully validated instrument. You have responded to most prior comments by clarifying terminology, adding aims and hypotheses, reorganizing the manuscript, expanding the discussion of BAQ/PSST, and scaling back some applied claims. However, I would still recommend **minor revision** . The main reason is that the manuscript continues to use some language that implies stronger validation and practical readiness than the data support, and several reporting/statistical details need clarification. Please see below for detailed comments.

Please submit your revised manuscript by Jul 02 2026 11:59PM. If you will need more time than this to complete your revisions, please reply to this message or contact the journal office at plosone@plos.org. When you're ready to submit your revision, log on to https://www.editorialmanager.com/pone/ and select the 'Submissions Needing Revision' folder to locate your manuscript file.

Please include the following items when submitting your revised manuscript:

- A letter that responds to each point raised by the academic editor and reviewer(s). You should upload this letter as a separate file labeled 'Response to Reviewers'.
- A marked-up copy of your manuscript that highlights changes made to the original version. You should upload this as a separate file labeled 'Revised Manuscript with Track Changes'.
- An unmarked version of your revised paper without tracked changes. You should upload this as a separate file labeled 'Manuscript'.

If you would like to make changes to your financial disclosure, please include your updated statement in your cover letter. Guidelines for resubmitting your figure files are available below the reviewer comments at the end of this letter.

If applicable, we recommend that you deposit your laboratory protocols in protocols.io to enhance the reproducibility of your results. Protocols.io assigns your protocol its own identifier (DOI) so that it can be cited independently in the future. For instructions see: https://journals.plos.org/plosone/s/submission-guidelines#loc-laboratory-protocols. Additionally, PLOS ONE offers an option for publishing peer-reviewed Lab Protocol articles, which describe protocols hosted on protocols.io. Read more information on sharing protocols at https://plos.org/protocols?utm_medium=editorial-email&utm_source=authorletters&utm_campaign=protocols.

As the corresponding author, your ORCID iD is verified in the submission system and will appear in the published article. PLOS supports the use of ORCID, and we encourage all coauthors to register for an ORCID iD and use it as well. Please encourage your coauthors to verify their ORCID iD within the submission system before final acceptance, as unverified ORCID iDs will not appear in the published article. *Only*  the individual author can complete the verification step; PLOS staff *cannot*  verify ORCID iDs on behalf of authors.

We look forward to receiving your revised manuscript.

Kind regards,

Domna Banakou

Academic Editor

PLOS One

Journal Requirements:

1. If the reviewer comments include a recommendation to cite specific previously published works, please review and evaluate these publications to determine whether they are relevant and should be cited. There is no requirement to cite these works unless the editor has indicated otherwise.

2. Please review your reference list to ensure that it is complete and correct. If you have cited papers that have been retracted, please include the rationale for doing so in the manuscript text, or remove these references and replace them with relevant current references. Any changes to the reference list should be mentioned in the rebuttal letter that accompanies your revised manuscript. If you need to cite a retracted article, indicate the article’s retracted status in the References list and also include a citation and full reference for the retraction notice.

Additional Editor Comments:

1. The Introduction is clear and coherent, but there are some minor issues. Specifically, the claim that "no measure exists to track the salience, valence, ..." should remain carefully qualified. Even if no comprehensive female-specific measure exists in the way you define it, the phrase "no measure exists" is broad. I would suggest changing it to something like "To our knowledge, no comprehensive female-specific measure currently assesses the salience, ...". Second, you say the study seeks to "validate" factors, but given that the work is explicitly formative, different wording such as "evaluate, refine, or provide preliminary support for" would be more accurate. Third, the a priori hypothesis of four broad domains is helpful, but the actual factor solution is much more granular. The Results/Discussion should explain that the final factor structure only partially aligned with the original broad conceptual domains.

2. A minor concern for clarification is in the methodology is regarding the exercise duration Lines 137-140 you state that duration intervals included 21-30 minutes, but then you say 20-30 min=35 min. That midpoint seems incorrect for either 20–30 or 21–30. Shouldn't it be 25 or 25.5, not 35? This should be corrected or clarified because exercise exposure variables were used in SEM models.

3. In the eligibility criteria you state "regular menstrual cycle between 25–35 days", but this may conflict conceptually with hormonal contraceptive users, depending on contraceptive type and withdrawal bleeding patterns. You should clarify how "regular menstrual cycle" was defined for HC users for example. Similarly, the limitations should be revised to avoid overgeneralizing.

4. The item-development process is clear and the process is suitable for early-stage scale development, but it remains partly subjective. This is acceptable as long as you frame the output as preliminary. In the manuscript you should better separate (a) pre-data conceptual item reduction, such as removal of synonymous or confusing terms, (b) data-driven reduction, such as removal based on unclear ratings, endorsement, and clustering, and (c) post hoc conceptual refinement, such as removal for redundancy, emotional/cognitive interpretation, or conceptual misalignment. Currently, you include these components but the manuscript could be more transparent about exactly how many items were removed at each post hoc stage and whether the final items or the conclusion's unpleasant and pleasant items are accurate. Table 5 reports 49 unpleasant items and 24 pleasant items, while the conclusion states that next steps would translate the "final list of 53 unpleasant and 28 pleasant items" into practical subscale scores. This appears inconsistent. The abstract also refers to present somatic items and factors, but the final number of retained items should be internally consistent across Results, Abstract, Discussion, and Conclusion.

5. Regarding the statistics, you provide a useful explanation of PROC VARCLUS, which is defensible for initial item reduction, especially with many items, but this is not a standard replacement for exploratory factor analysis or modern psychometric item-response methods. You should justify more clearly why PROC VARCLUS was preferred over EFA, parallel analysis, exploratory structural equation modeling, or other scale-development approaches. Additionally, you used multiple imputation via the EM algorithm because PROC VARCLUS cannot accommodate missing data. However, more information is needed on the missingness pattern and the assumptions underlying imputation. Since participants could mark items as "Unclear" missingness may be informative rather than missing at random.

6. The CFA section is improved, but the model fit interpretation needs correction (or softening). You state that the unpleasant model showed good fit based on RMSEA/SRMR and acceptable fit by CFI/TLI. Yet CFI/TLI values are around 0.85/0.84 for unpleasant and 0.90/0.89 for pleasant in the earlier table. These are typically below conventional “acceptable” thresholds, especially since the Methods cite desired values of CFI/TLI ≥ .95. The clean manuscript says the model fit indices were moved to S1 Table 2, but the issue remains: if the actual CFI/TLI values are below the stated thresholds, the manuscript should say so directly. Furthermore, you should report whether SEM p-values were corrected for multiple testing or explicitly state they were exploratory and uncorrected. Also, you should clarify directionality in the SEM models. The text sometimes says factors were associated with BAQ/PSST and behaviors, while elsewhere it says variables "predicted" factors or that factors were "related to" variables.

7. Some labels are physiologically loaded, especially "Parasympathetic Activation", without direct physiological measurement. Also, you should use "female, women, and female-specific" carefully. Participants are described as female university students, but the inventory is framed as about women. You may want to state whether "female" refers to sex assigned at birth, gender identity, or eligibility wording used in recruitment/screening.

8. Lines 337-338, the phrase "validated and refined with CFA" should be softened because this is not independent validation.

[Note: HTML markup is below. Please do not edit.]

Reviewers' comments:

Reviewer's Responses to Questions

**Comments to the Author**

1. If the authors have adequately addressed your comments raised in a previous round of review and you feel that this manuscript is now acceptable for publication, you may indicate that here to bypass the “Comments to the Author” section, enter your conflict of interest statement in the “Confidential to Editor” section, and submit your "Accept" recommendation.

Reviewer #1: All comments have been addressed

**********

2. Is the manuscript technically sound, and do the data support the conclusions?

The manuscript must describe a technically sound piece of scientific research with data that supports the conclusions. Experiments must have been conducted rigorously, with appropriate controls, replication, and sample sizes. The conclusions must be drawn appropriately based on the data presented.

Reviewer #1: Yes

**********

3. Has the statistical analysis been performed appropriately and rigorously?

Reviewer #1: Yes

**********

4. Have the authors made all data underlying the findings in their manuscript fully available?

The PLOS Data policy requires authors to make all data underlying the findings described in their manuscript fully available without restriction, with rare exception (please refer to the Data Availability Statement in the manuscript PDF file). The data should be provided as part of the manuscript or its supporting information, or deposited to a public repository. For example, in addition to summary statistics, the data points behind means, medians and variance measures should be available. If there are restrictions on publicly sharing data—e.g. participant privacy or use of data from a third party—those must be specified.

Reviewer #1: Yes

**********

5. Is the manuscript presented in an intelligible fashion and written in standard English?

PLOS ONE does not copyedit accepted manuscripts, so the language in submitted articles must be clear, correct, and unambiguous. Any typographical or grammatical errors should be corrected at revision, so please note any specific errors here.

Reviewer #1: Yes

**********

6. Review Comments to the Author

Please use the space provided to explain your answers to the questions above. You may also include additional comments for the author, including concerns about dual publication, research ethics, or publication ethics. (Please upload your review as an attachment if it exceeds 20,000 characters)

Reviewer #1: I would like to thank the authors for conducting the revision process with such care and thoroughness. I believe that this manuscript, which already showed strong potential in its original form, has now reached a level of quality that makes it suitable for publication in its current version. I would also like to sincerely thank the Editor and the Journal for giving me the opportunity to review this interesting and valuable work.

**********

7. PLOS authors have the option to publish the peer review history of their article (what does this mean?). If published, this will include your full peer review and any attached files.

If you choose “no”, your identity will remain anonymous but your review may still be made public.

**Do you want your identity to be public for this peer review?**  For information about this choice, including consent withdrawal, please see our Privacy Policy.

Reviewer #1: No

**********

[NOTE: If reviewer comments were submitted as an attachment file, they will be attached to this email and accessible via the submission site. Please log into your account, locate the manuscript record, and check for the action link "View Attachments". If this link does not appear, there are no attachment files.]

To ensure your figures meet our technical requirements, please review our figure guidelines: https://journals.plos.org/plosone/s/figures

You may also use PLOS’s free figure tool, NAAS, to help you prepare publication quality figures: https://journals.plos.org/plosone/s/figures#loc-tools-for-figure-preparation.

NAAS will assess whether your figures meet our technical requirements by comparing each figure against our figure specifications.

# Author comment: Author response to Decision Letter 3

**DOI:** 10.1371/journal.pone.0353167.r006

16 Jun 2026

Journal Requirements:

1. If the reviewer comments include a recommendation to cite specific previously published works, please review and evaluate these publications to determine whether they are relevant and should be cited. There is no requirement to cite these works unless the editor has indicated otherwise.

N/A

2. Please review your reference list to ensure that it is complete and correct. If you have cited papers that have been retracted, please include the rationale for doing so in the manuscript text, or remove these references and replace them with relevant current references. Any changes to the reference list should be mentioned in the rebuttal letter that accompanies your revised manuscript. If you need to cite a retracted article, indicate the article’s retracted status in the References list and also include a citation and full reference for the retraction notice.

All references were reviewed; none were retracted.

Additional Editor Comments:

** NOTE: all line numbers mentioned below refer to line numbers of the ‘clean’ manuscript.

1. The Introduction is clear and coherent, but there are some minor issues. Specifically, the claim that "no measure exists to track the salience, valence, ..." should remain carefully qualified. Even if no comprehensive female-specific measure exists in the way you define it, the phrase "no measure exists" is broad. I would suggest changing it to something like "To our knowledge, no comprehensive female-specific measure currently assesses the salience, ...".

The proposed edit to the “no… measure exists” statement has been made (line 42-43)

Second, you say the study seeks to "validate" factors, but given that the work is explicitly formative, different wording such as "evaluate, refine, or provide preliminary support for" would be more accurate.

The term “validated” was removed (lined 84) as the next sentence makes the point more clearly (“proof of concept”) and is more aligned with the editor’s point. Relatedly, “validate” was also mentioned on line 224 (see Comment 8); we changed this to “confirmed” (with CFA).

Third, the a priori hypothesis of four broad domains is helpful, but the actual factor solution is much more granular. The Results/Discussion should explain that the final factor structure only partially aligned with the original broad conceptual domains.

We have added a statement to this effect in the first paragraph of the discussion (lines 484-486). This factor structure partially aligned with our hypothesis of four broad-valence factors (kinesthetic, energetic, visceral, wellness) but was far more nuanced: factors were valence-specific and often reflected integrated physiological states.

2. A minor concern for clarification is in the methodology is regarding the exercise duration Lines 137-140 you state that duration intervals included 21-30 minutes, but then you say 20-30 min=35 min. That midpoint seems incorrect for either 20–30 or 21–30. Shouldn't it be 25 or 25.5, not 35? This should be corrected or clarified because exercise exposure variables were used in SEM models.

Thank you for noticing this; it has been corrected to 25 min (line 141).

3. In the eligibility criteria you state "regular menstrual cycle between 25–35 days", but this may conflict conceptually with hormonal contraceptive users, depending on contraceptive type and withdrawal bleeding patterns. You should clarify how "regular menstrual cycle" was defined for HC users for example. Similarly, the limitations should be revised to avoid overgeneralizing.

The criteria “regular menstrual cycle between 25-35 days” was used for all participants. HC use was information collected during the study period; it was not an eligibility criterion. We have added a clarifying point to this effect: It is noteworthy that all participants in the HC and NC groups reported 25-35 day cycles; thus, this observation is not related to the absence of menstrual bleeding. (Line 593-594)

4. The item-development process is clear and the process is suitable for early-stage scale development, but it remains partly subjective. This is acceptable as long as you frame the output as preliminary. In the manuscript you should better separate (a) pre-data conceptual item reduction, such as removal of synonymous or confusing terms, (b) data-driven reduction, such as removal based on unclear ratings, endorsement, and clustering, and (c) post hoc conceptual refinement, such as removal for redundancy, emotional/cognitive interpretation, or conceptual misalignment.

We added the following qualifying phrases in the Results: “pre-data conceptual item reduction step” to describe the first data reduction phase (line 264), a “post-data conceptual item reduction step’ to describe a second data reduction step that occurred using the raw/un-modeled data (line 271). Then, we describe “a data-driven item reduction step… ” on line 289 (unpleasant) and line 338 (pleasant) and “post hoc conceptual refinement step” to lines 307 (unpleasant items) & lines 354-355 (pleasant items) of the Results. We also added this terminology to the Limitations section of the Discussion (lines 655-656).

Currently, you include these components but the manuscript could be more transparent about exactly how many items were removed at each post hoc stage and whether the final items or the conclusion's unpleasant and pleasant items are accurate. Table 5 reports 49 unpleasant items and 24 pleasant items, while the conclusion states that next steps would translate the "final list of 53 unpleasant and 28 pleasant items" into practical subscale scores. This appears inconsistent. The abstract also refers to present somatic items and factors, but the final number of retained items should be internally consistent across Results, Abstract, Discussion, and Conclusion.

We carefully reviewed the item reduction process. We corrected disparate numbers throughout the text. The abstract clarifies the final 73 items and 13 factors (line 20), with matches with the Conclusion item numbers (line 684). This aligns with the S2 Appendix pilot inventory form as well. We added details along with the above-suggested item reduction step terminology in the Results to clarify when/why items were trimmed (bulleted list summary below).

The unpleasant data-driven steps started with 120 items (line 290-315)

- 30 synonymous items

- 10 poorly loading items

80 items retained during first-order clustering were then subjected to post hoc conceptual refinement

- 8 items from first-order clusters that couldn’t be combined with other clusters in second-order clustering process

- 5 items that were redundant but not identified as synonymous in first-order clustering

- 6 items that had cognitive or emotional interpretations

- 8 items that did not align with the second-order cluster conceptual themes

53 items that loaded onto 9 factors were retained (line 316-317).

- 4 additional items were removed during the CFA

THE PILOT INVENTORY PROPOSES 49 ITEMS FOR 9 FACTORS (line 326)

The pleasant data-driven steps started with 55 items (line 343-355)

- 16 synonymous or poorly loading items

39 items retained during first-order clustering were then subjected to post hoc conceptual refinement

- 1 item from first-order clusters that couldn’t be combined with other clusters in second-order clustering process

- 7 items that were redundant but not identified as synonymous in first-order clustering

- 3 items that did not align with the second-order cluster conceptual themes

28 items that loaded onto 4 factors were retained (line 356-357).

- 4 additional items were removed during the CFA

PILOT INVENTORY PROPOSES 24 ITEMS FOR 4 FACTORS (line 363)

5. Regarding the statistics, you provide a useful explanation of PROC VARCLUS, which is defensible for initial item reduction, especially with many items, but this is not a standard replacement for exploratory factor analysis or modern psychometric item-response methods. You should justify more clearly why PROC VARCLUS was preferred over EFA, parallel analysis, exploratory structural equation modeling, or other scale-development approaches.

We acknowledge that PROC VARCLUS is a less common approach that was necessitated based on the data structure and the early development stage of analyses (lines 184-187). Initially, we tried EFA, CFA & PCA, but the number of items and novelty of the content made obtaining an intuitive and concise model difficult. Instead, we modeled our approach after the MAIA development process as those authors provided an excellent description of the PROC VARCLUS steps they used to successfully develop their scale. This methodology is preferrable over other exploratory data reduction tools (e.g., exploratory factor analysis) during survey development stages as it superior when there are a large number of items and discrete, iterative pruning steps are needed to characterize theoretically-sound clusters.

Additionally, you used multiple imputation via the EM algorithm because PROC VARCLUS cannot accommodate missing data. However, more information is needed on the missingness pattern and the assumptions underlying imputation. Since participants could mark items as "Unclear" missingness may be informative rather than missing at random.

To perform the imputation, we assumed data were MAR; however, we did not explicitly test this (we now note this in Limitations, line 653-654). We also clarify that we removed items with large numbers of “Unclear” ratings prior to the imputation (line 189-190). As a result, for the PROC VARCLUS, the number of “unclear” ratings that were converted to missing for imputation were low (80% of retained items had <10 “unclear” ratings).

6. The CFA section is improved, but the model fit interpretation needs correction (or softening). You state that the unpleasant model showed good fit based on RMSEA/SRMR and acceptable fit by CFI/TLI. Yet CFI/TLI values are around 0.85/0.84 for unpleasant and 0.90/0.89 for pleasant in the earlier table. These are typically below conventional “acceptable” thresholds, especially since the Methods cite desired values of CFI/TLI ≥ .95. The clean manuscript says the model fit indices were moved to S1 Table 2, but the issue remains: if the actual CFI/TLI values are below the stated thresholds, the manuscript should say so directly.

Yes, these statements were not correct; they should have aligned with the MAIA scale development process (see lines 234-235). We also added a statement in Limitations (lines 654-659) directly addressing the low CFI/TLI values. Further, the CFI and TLI values for the CFA models for the unpleasant factors (and TLI for the pleasant factors) were below the range typically considered acceptable. Thus, results should be replicated in other samples and undergo additional rigorous psychometric and validity testing. However, considering the preliminary nature and iterative strategies used of inventory development, we propose that the pilot inventory (S2 Appendix File) may be a useful starting point for further study.

Furthermore, you should report whether SEM p-values were corrected for multiple testing or explicitly state they were exploratory and uncorrected.

This has been correct on lines 249-250. Analyses were uncorrected for multiple testing due to exploratory nature of study.

Also, you should clarify directionality in the SEM models. The text sometimes says factors were associated with BAQ/PSST and behaviors, while elsewhere it says variables "predicted" factors or that factors were "related to" variables.

We were unable to locate the word “predicted” in the clean version but agree that it should be purged as all analyses were associative and non-directional. Per “associated” versus “related to” - both terms are non-directional but for clarity we have adjusted all statements pertaining to the factors and validated surveys using the term: associated with (lines 96, 425-470, 484, 587-588, 603, 608, 615-625, 641).

7. Some labels are physiologically loaded, especially "Parasympathetic Activation", without direct physiological measurement.

We acknowledge the lack of physiological validation of the scales (we are actually preliminary a physiology lab) and have noted this in the limitations (line 668-670). The names given to the identified factors may require additional refinement (e.g., Heat & Urgency Responses) and/or validation with physiological data (e.g., Parasympathetic Activation).

Also, you should use "female, women, and female-specific" carefully. Participants are described as female university students, but the inventory is framed as about women. You may want to state whether "female" refers to sex assigned at birth, gender identity, or eligibility wording used in recruitment/screening.

We did attempt to be thoughtful throughout this process in considering whether to use the word “female” or “women”. The study from which these data are drawn included only biological females – because the inclusion criteria required a 25-35 day menstrual cycle (line 104). Moreover, the broader study goals focused on menstrual health, which is a biological-female-specific health issue. HOWEVER, as the survey developed, we recognized that the inventory may be useful and appropriate for a broader spectrum of individuals who identify as women. This, however, would require validation with appropriate samples once the inventory is finalized and is outside the scope of this manuscript. That said, we went back through the document and removed the term “female” except to describe the sample or in relation to menstrual and reproductive health.

8. Lines 337-338, the phrase "validated and refined with CFA" should be softened because this is not independent validation.

This has been corrected to “confirmed and refined” (see Comment 1 response C above).

# Editor report: Decision Letter 3

**DOI:** 10.1371/journal.pone.0353167.r007

**Authors:** Stefano Federici, Domna Banakou, Domna Banakou, Domna Banakou

21 Jun 2026

Capturing women's bodily experiences: Conception and formative stages of the Women's Somatic Experience Inventory

PONE-D-25-18681R3

Dear Dr. Piersol,

We’re pleased to inform you that your manuscript has been judged scientifically suitable for publication and will be formally accepted for publication once it meets all outstanding technical requirements.

Within one week, you’ll receive an e-mail detailing the required amendments. When these have been addressed, you’ll receive a formal acceptance letter and your manuscript will be scheduled for publication.

An invoice will be generated when your article is formally accepted. Please note, if your institution has a publishing partnership with PLOS and your article meets the relevant criteria, all or part of your publication costs will be covered. Please make sure your user information is up-to-date by logging into Editorial Manager at Editorial Manager® and clicking the ‘Update My Information' link at the top of the page. For questions related to billing, please contact billing support.

If your institution or institutions have a press office, please notify them about your upcoming paper to help maximize its impact. If they’ll be preparing press materials, please inform our press team as soon as possible -- no later than 48 hours after receiving the formal acceptance. Your manuscript will remain under strict press embargo until 2 pm Eastern Time on the date of publication. For more information, please contact onepress@plos.org.

Kind regards,

Domna Banakou

Academic Editor

PLOS One

Additional Editor Comments (optional):

Reviewers' comments:

# Editor report: Acceptance letter

**DOI:** 10.1371/journal.pone.0353167.r008

**Authors:** Stefano Federici, Domna Banakou, Domna Banakou, Domna Banakou

PONE-D-25-18681R3

PLOS One

Dear Dr. Piersol,

I'm pleased to inform you that your manuscript has been deemed suitable for publication in PLOS One. Congratulations! Your manuscript is now being handed over to our production team.

At this stage, our production department will prepare your paper for publication. This includes ensuring the following:

* All references, tables, and figures are properly cited

* All relevant supporting information is included in the manuscript submission,

* There are no issues that prevent the paper from being properly typeset

You will receive further instructions from the production team, including instructions on how to review your proof when it is ready. Please keep in mind that we are working through a large volume of accepted articles, so please give us a few days to review your paper and let you know the next and final steps.

Lastly, if your institution or institutions have a press office, please let them know about your upcoming paper now to help maximize its impact. If they'll be preparing press materials, please inform our press team within the next 48 hours. Your manuscript will remain under strict press embargo until 2 pm Eastern Time on the date of publication. For more information, please contact onepress@plos.org.

You will receive an invoice from PLOS for your publication fee after your manuscript has reached the completed accept phase. If you receive an email requesting payment before acceptance or for any other service, this may be a phishing scheme. Learn how to identify phishing emails and protect your accounts at https://explore.plos.org/phishing.

If we can help with anything else, please email us at customercare@plos.org.

Thank you for submitting your work to PLOS ONE and supporting open access.

Kind regards,

PLOS ONE Editorial Office Staff

on behalf of

Dr. Domna Banakou

Academic Editor

PLOS One
